# Integration of transcription regulation and functional genomic data reveals lncRNA SNHG6’s role in hematopoietic differentiation and leukemia

**DOI:** 10.1186/s12929-024-01015-8

**Published:** 2024-02-28

**Authors:** Joshua M. Hazan, Raziel Amador, Tahleel Ali-Nasser, Tamar Lahav, Stav Roni Shotan, Miryam Steinberg, Ziv Cohen, Dvir Aran, David Meiri, Yehuda G. Assaraf, Roderic Guigó, Assaf C. Bester

**Affiliations:** 1 Department of Biology, Technion-Israel Institute of Technology, 3200003 Haifa, Israel; 2 Centre for Genomic Regulation (CRG), Doctor Aiguader 88, 08003 Barcelona, Catalonia Spain; 3 Universitat de Barcelona (UB), Barcelona, Catalonia Spain; 4 The Taub Faculty of Computer Science, Technion-Israel Institute of Technology, 3200003 Haifa, Israel; 5 The Fred Wyszkowski Cancer Research Laboratory, Department of Biology, Technion-Israel Institute of Technology, 3200003 Haifa, Israel; 6 Universitat Pompeu Fabra (UPF), Barcelona, Catalonia Spain

**Keywords:** lncRNA, CRISPR screening, Machine learning, Gene regulation, Hematopoiesis.

## Abstract

**Background:**

Long non-coding RNAs (lncRNAs) are pivotal players in cellular processes, and their unique cell-type specific expression patterns render them attractive biomarkers and therapeutic targets. Yet, the functional roles of most lncRNAs remain enigmatic. To address the need to identify new druggable lncRNAs, we developed a comprehensive approach integrating transcription factor binding data with other genetic features to generate a machine learning model, which we have called INFLAMeR (Identifying Novel Functional LncRNAs with Advanced Machine Learning Resources).

**Methods:**

INFLAMeR was trained on high-throughput CRISPR interference (CRISPRi) screens across seven cell lines, and the algorithm was based on 71 genetic features. To validate the predictions, we selected candidate lncRNAs in the human K562 leukemia cell line and determined the impact of their knockdown (KD) on cell proliferation and chemotherapeutic drug response. We further performed transcriptomic analysis for candidate genes. Based on these findings, we assessed the lncRNA small nucleolar RNA host gene 6 (*SNHG6*) for its role in myeloid differentiation. Finally, we established a mouse K562 leukemia xenograft model to determine whether *SNHG6* KD attenuates tumor growth in vivo.

**Results:**

The INFLAMeR model successfully reconstituted CRISPRi screening data and predicted functional lncRNAs that were previously overlooked. Intensive cell-based and transcriptomic validation of nearly fifty genes in K562 revealed cell type-specific functionality for 85% of the predicted lncRNAs. In this respect, our cell-based and transcriptomic analyses predicted a role for *SNHG6* in hematopoiesis and leukemia. Consistent with its predicted role in hematopoietic differentiation, *SNHG6* transcription is regulated by hematopoiesis-associated transcription factors. *SNHG6* KD reduced the proliferation of leukemia cells and sensitized them to differentiation. Treatment of K562 leukemic cells with hemin and PMA, respectively, demonstrated that *SNHG6* inhibits red blood cell differentiation but strongly promotes megakaryocyte differentiation. Using a xenograft mouse model, we demonstrate that *SNHG6* KD attenuated tumor growth in vivo.

**Conclusions:**

Our approach not only improved the identification and characterization of functional lncRNAs through genomic approaches in a cell type-specific manner, but also identified new lncRNAs with roles in hematopoiesis and leukemia. Such approaches can be readily applied to identify novel targets for precision medicine.

**Supplementary Information:**

The online version contains supplementary material available at 10.1186/s12929-024-01015-8.

## Background

Long non-coding RNAs (lncRNAs) are RNA transcripts that exceed 200 nucleotides in length and have low or no protein-coding potential [1–3]. The transcription of most lncRNAs is regulated through the same mechanism as that of protein-coding genes (PCGs), involving RNA polymerase II and transcription factors (TFs). Furthermore, lncRNAs share several characteristics with PCGs, including a poly-A tail and gene bodies consisting of exons and introns. They comprise one of the largest groups of non-coding elements in the human genome, with the estimated number of annotated lncRNAs ranging from 15,000 to 150,000 genes [1–4]. Despite this, lncRNAs are one of the most poorly understood groups of non-coding elements in terms of functional characterization [5, 6]. Identifying functional lncRNAs remains a challenge due to experimental limitations and an incomplete understanding of their biology.

Experimentally, the functionality of large groups of lncRNAs can be studied through reverse genetic screenings. Library-based screens using various gene perturbation methods have been performed, including antisense oligonucleotides [7], short hairpin RNA with engineered siRNAs [8], and CRISPR-Cas9 [1, 9]. While CRISPR has revolutionized reverse genetics by increasing accuracy for the study of PCGs, Cas9-based knockout is not effective for lncRNAs, which are minimally sequence-dependent. Nevertheless, several library screens have been performed using this method, typically by targeting splice sites [10] or with paired gRNAs designed to remove entire exons, introns, promoters, or transcription start sites (TSSs) from the genome [1, 11, 12]. Alternative CRISPR-based tools have been developed to modulate gene expression without modifying the genome by using a nuclease-dead Cas9 (dCas9) that binds the target sequence without cleaving the DNA. This can be combined with repressive effector protein domains for CRISPR inhibition (CRISPRi) [13] or with transcription activating domains for CRISPR activation (CRISPRa) [14, 15]. Additionally, other CRISPR-based approaches are under development, such as stable CRISPR epigenetic silencing (CRISPR-off) and Cas13, which targets RNA [16]. However, screening approaches are limited to specific cell lines and tend to suffer from low sensitivity and accuracy [1].

Computational methods have emerged as a promising approach for predicting the functionality of lncRNAs. These methods include investigating evolutionary conservation [17] and sequence similarities [18], as well as considering expression, differential expression, or co-expression in specific biological contexts [19]. In recent years, multi-omics data integration has been used to complement these approaches and improve their accuracy [20].

Recent advances in machine learning (ML) techniques show great potential for adaptation to address this complex problem. Supervised ML is used for classifying elements based on highly complex data, high dimensionality, or high volumes of data. Indeed, by integrating multiple sources of information, ML models have already been used to successfully predict complex regulatory elements such as enhancers [21], as well as to identify gene regulatory networks and the functional roles of non-coding RNAs in gene regulation. While ML has successfully distinguished lncRNAs from mRNAs [22] and classified tumors based on lncRNA expression [23], predicting the functionality and sub-classification of lncRNAs remains a challenging task. Identifying meaningful features and reliable training sets is essential for classifying functional lncRNAs in specific biological contexts.

To bridge this gap, we developed an ML model known as INFLAMeR (Identifying Novel Functional LncRNAs with Advanced Machine Learning Resources). The model was based on 143 genetic features that focus on cell type-specific regulatory mechanisms and was trained on previous CRISPRi screens [24–26]. INFLAMeR successfully replicated the CRISPRi screening results and identified additional lncRNAs with high prediction scores. Through cell-based and transcriptomics analyses, we tested the true positive and true negative accuracy of INFLAMeR’s predictions for more than forty lncRNAs in vitro. Experimental validation based on the INFLAMeR scores demonstrated high predictive accuracy. Of all the lncRNAs with high INFLAMeR scores, 85% displayed significant phenotypic impacts upon KD, whereas lncRNAs with low INFLAMeR scores did not show any effects in the same assays. We further characterized the lncRNA small nucleolar host gene 6 (*SNHG6*) and uncovered its role in hematopoietic differentiation. Overall, our results indicate that INFLAMeR enhances the prediction capacity of functional lncRNAs to a greater extent than large-scale screening assays and can identify lncRNAs for characterization in a cell-type-specific manner.

## Methods

### Developing the ML algorithm

#### ENCODE TF ChIP-seq

We used ENCODE TF ChIP-seq data to determine TF peak height within lncRNA promoters across five cell lines; HEK293T, HeLa, MCF7, K562, and H1-hESC; using 124 TFs.

We downloaded the bigBed narrowPeak files with optimal irreproducible discovery rate thresholded peaks in *hg19* assembly coordinates. We applied a window of [− 300; + 100] bp surrounding the TSS to obtain lncRNA promoters, as described previously [27]. Next, using *BEDTools* intersect version: 2.27, TFs bigBed, and lncRNA promoters bed files, the TF peak height was obtained. A 10% intersection cutoff between TF ChIP-seq and lncRNA promoter was used.

#### Model training

Stratified tenfold cross-validation with three different randomizations in each repetition was adopted to train all supervised models, using the *RepeatedStratifiedKFold* class from *scikit-learn* version 0.24.1, with 90% and 10% for training and test, respectively (Additional file 1: Fig. S1a).

#### XGBoost

The *dmlc XGBoost* library (https://xgboost.readthedocs.io/en/latest/index.html) version 1.3.3 was used for implementing the XGBoost model [28]. XGBoost is a type of gradient boosting decision tree method.

As our dataset was unbalanced, the ratio of the minority positive class (hits) versus the majority negative class (not hits) was 1:55; we adopted the XGBoost *scale position weight* parameter to train a cost-sensitive XGBoost classifier for imbalanced data. The default *scale position weight*: 54.81 [sum(majority negative class)/sum(minority positive class)], 100, and 1000 values; in addition to other XGBoost hyper-parameters, were used for grid search coupled with stratified tenfold cross-validation. XGBoost was tuned to search for an optimal sensitivity and specificity solution. To tune the hyper-parameters, we adopted the *GridSearchCV scikit-learn* class to improve the performance of the model, using a NVIDIA GPU GeForce RTX-2060 (drivers version 465.31, and CUDA version 11.3). The hyper-parameters tuned for XGBoost and their final values are displayed below (Additional file 1: Fig. S1b):

- Scale position weight: 100
- Learning rate: 0.05
- Max depth: 5
- Regularization lambda: 5.0
- Gamma: 1.0

#### Metrics

To evaluate model performance, we determined the sensitivity (recall), specificity, precision, F1-score, area under the receiver operating characteristic curve (AUROC), AUPRC, Brier score, and Brier skill score, using the test set and the stratified tenfold cross-validation process described above.

#### Recursive feature elimination (RFE)

Recursive feature elimination (RFE)—removing the least important features based on SHAP values with stratified cross-validation—was implemented using the *ShapRFECV* class from the *probatus* python module (https://ing-bank.github.io/probatus/index.html), version 1.8.4. The step was one feature per iteration, with sensitivity and specificity as scoring metrics.

#### Model explainability

*TreeExplainer* from the SHapley Additive eXPlanations (SHAP) framework version 0.39.0 was used to explain the output of our XGBoost model. Global and local explanations were obtained based on the complete dataset. The SHAP framework is based on Shapley values, which is a cooperative game theory concept [29].

#### Computational settings

All the computational analyses for this section were carried out using Linux-based distributions and Python 3.8.5, with computational resources provided by the Centre for Genomic Regulation (CRG), Spain.

The raw features data used to train the ML can be found in Additional file 2: Table S1.

The code for the INFLAMeR algorithm can be found at the following GitHub repository: https://github.com/razielar/INFLAMer.

### Cell culture

The following cell lines were used in the current study: K562 chronic myeloid leukemia cells and HEK293T human embryonic kidney cells. K562 cells were maintained in RPMI-1640 medium containing 1% l-glutamine and 25 mM HEPES pH 7.4 (Gibco, Waltham, MA, USA), supplemented with 10% fetal calf serum (FCS; Gibco) and 1% penicillin–streptomycin solution (Sartorius, Goettingen, Germany) and passaged every 2–4 days. HEK293T cells were maintained in Dulbecco’s Modified Eagle’s Medium (DMEM; Rhenium, Modiin, Israel) supplemented with 10% FCS, 1% penicillin–streptomycin solution, and 1% l-glutamine (Sartorius) and passaged every 2–4 days.

### Stable knockdown of target lncRNAs in K562 cells

For KD by CRISPRi, K562 cells were stably transduced with the pHR-SFFV-dCas9-BFP-KRAB cassette (kindly provided by Stanley Qi & Jonathan Weissman; Addgene plasmid #46911). Transduction was performed via lentiviral delivery using PolyJet In Vitro Transfection Reagent (SignaGen, Frederick, MD, USA) following the manufacturer’s protocol. Briefly, HEK293T cells were seeded 18–24 h before transfection to obtain a cell confluency of ~ 90% at the time of transfection. The cells were transfected with the plasmid of interest, as well as the VSV-G (Addgene plasmid #8454) and psPAX2 (Addgene plasmid #12260) lentiviral packaging plasmids. Lentivirus was harvested 48–72 h after transfection. Lentiviral transduction was performed by incubating K562 cells with DMEM containing the harvested lentivirus for 24 h; next, the media was replaced with fresh RPMI-1640 growth medium, and cells were allowed to recover for 48–72 h. Finally, cells stably expressing blue fluorescent protein (BFP) were selected by fluorescence-activated cell sorting (FACS) using the BD FACSAria-IIIu (BD Biosciences, Franklin Lakes, NJ, USA) to obtain the K562-CRISPRi stable cell line.

The gRNAs used for KD were obtained from a previous lncRNA-specific gRNA library [24]. The gRNAs were cloned into either pCRISPRia-v2 (kindly provided by Jonathan Weissman; Addgene plasmid #84832) or pSB700 expressing blasticidin resistance (pSB700-Blast; kindly provided by George Church; Addgene plasmid #64046). To confirm successful incorporation of the insert, *E. coli* DH5α colonies transformed with the cloned plasmids by heat-shock were subjected to colony PCR using three primers: two primers surrounding the insert site and a third primer overlapping the sequence that is removed during cloning. The PCR products were visualized after 2% agarose gel electrophoresis at 160 V for 30 min. The backbone plasmid with no insert showed two bands of 800 bp and 500 bp for pCRISPRia-v2, or 600 bp and 150 bp for pSB700; plasmids with successful incorporation of the insert showed the larger band only. The primers used to confirm successful ligation can be found in Additional file 3: Table S2.

The gRNAs were stably transduced into K562-CRISPRi cells via lentiviral transduction as described above. For each lncRNA, two gRNAs targeting the same gene were simultaneously transduced. The first gRNA was cloned into pCRISPRia-v2 (expressing tagBFP and puromycin resistance), and the second gRNA was cloned into pSB700-Blast. After incubating the cells with growth medium containing the harvested lentivirus for 24 h, the medium was replaced with fresh RPMI-1640 growth medium supplemented with 1 μg/ml puromycin and 10 μg/ml blasticidin every 48–72 h for 7–10 days. Additionally, cells were transduced with plasmids containing non-targeting gRNAs and subjected to antibiotic selection or selected by FACS. The sequences of the gRNAs used can be found in Additional file 4: Table S3.

### Stable knockout of target lncRNAs in K562 cells

To achieve functional knockout of the target lncRNAs, paired gRNAs were designed using the CRISPETa tool [30] to delete a region of approximately 500–1000 bp flanking the TSS of the target lncRNA. The first gRNA of each pair was cloned into lentiCRISPR v2 (kindly provided by Feng Zhang; Addgene plasmid #52961), and the second was cloned into pSB700-Blast. Successful incorporation of the insert was confirmed for pSB700-Blast as above; for lentiCRISPR v2, two primers were used surrounding the insert region. After colony PCR, the backbone plasmid with no insert showed a band of approximately 2300 bp, while plasmids with successful incorporation of the insert displayed a single band of approximately 500 bp. The primers used to confirm successful ligation can be found in Additional file 3: Table S2.

Each pair of gRNAs was transduced into naïve K562 cells as described above, and antibiotic selection was performed using puromycin and blasticidin as described above. The sequences of the gRNAs can be found in Additional file 4: Table S3.

### Rescue of lncRNA expression by stable plasmid overexpression

The expression of selected lncRNAs was rescued by stable transduction of the mature lncRNA sequence of each gene into cells with confirmed KD of the gene. The lncRNA sequences (provided in Additional file 5: Table S4) were synthesized by Twist Bioscience (San Francisco, CA, USA) in their Twist Cloning Vector. The sequences were then ligated into N174-MCS (kindly provided by Adam Karpf; Addgene plasmid #81061). Lentiviral transduction was performed as described above into K562-CRISPRi cells with confirmed KD of the same lncRNA, followed by antibiotic selection with 600 μg/ml Geneticin (G418; Invivogen, Toulouse, France) for 7–10 days.

### RNA isolation and RT-qPCR

Following stable cell transduction with the indicated gRNAs or plasmids, RNA extraction was performed using TRIzol reagent according to standard protocols. RNAs were reverse transcribed to cDNAs using the Quantabio qScript cDNA synthesis kit (QIAGEN, Beverley, MA, USA). The cDNAs were then used for qPCR using qPCRBIO SyGreen Blue Mix Lo-ROX (PCR Biosystems, London, UK), and expression was measured relative to that of cells transduced with a non-targeting sgRNA. Changes in gene expression were calculated using the standard 2^−ΔΔCt^ method and normalized to GAPDH and PGK1 [31]. The primers used for qPCR are listed in Additional file 3: Table S2.

### Two-color competitive cell growth assay

To determine the effect of KD on cell growth and proliferation, each sample was subjected to a two-color competitive cell growth (CCG) assay [24, 32] as follows. K562-CRISPRi cells transduced with a non-targeting sgRNA—expressing green fluorescent protein (GFP)—were co-seeded at 2 × 10^5^ cells/ml in the same well as cells transduced with sgRNAs targeting the indicated gene—expressing BFP—at 2 × 10^5^ cells/ml in 2 ml RPMI-1640 growth medium (day 0). The percentage of GFP- and BFP-expressing cells was determined every 2–4 days by flow cytometry for 14 days using the Agilent NovoCyte flow cytometer (Agilent, Santa Clara, CA, USA). Relative cell growth and proliferation was calculated as the proportion of BFP-expressing cells normalized to that at day 0.

### Cell cycle analysis

Approximately 10^6^ cells were harvested, washed in PBS, and fixed in 1 ml 70% ethanol for at least 2 h at 4 °C. The samples were then centrifuged at 400×*g* for 5 min, washed once in PBS, and incubated in 0.5 ml PBS containing 25 µg/ml propidium iodide solution (BioLegend, San Diego, CA, USA) and 10 µg/ml RNase I for 30 min in the dark, followed by flow cytometry analysis using the Agilent NovoCyte flow cytometer.

### Apoptosis analysis

Approximately 10^6^ cells were harvested, washed in PBS, and incubated in 200 µl Annexin V binding buffer containing 100 ng/ml APC-Annexin V (BioLegend, San Diego, CA, USA) and 5 µM SYTOX Green Nucleic Acid Stain (Invitrogen, Waltham, MA, USA) for 20 min in the dark, followed by flow cytometric analysis using the Agilent NovoCyte flow cytometer. APC-Annexin V was used to identify cells in early apoptosis, and SYTOX Green was used to identify cells in late apoptosis.

### Assessing DNA damage by western blot analysis

To investigate the impact of KD on DNA damage repair, we determined the levels of phospho-histone H2AX (γH2AX), an established marker of DNA damage [33]. Protein extraction was performed using the hot lysis method. Briefly, cells were rinsed in PBS and incubated in 200 µL hot lysis buffer—containing 10% SDS, 5 mM EDTA, and 50 mM Tris pH 7.5—for 15 min at 100 °C, followed by sonication with two 15-s pulses of 35% amplitude, centrifugation at 15,000×*g* and 12 °C for 20 min, and recovery of the supernatant. The protein concentration was determined using the standard BCA assay (Sigma-Aldrich, St. Louis, MO, USA), followed by 12% SDS-PAGE and western blotting as follows. Following SDS-PAGE, samples were transferred to LV-PVDF membranes, blocked with 5% skim milk in TBST, and incubated with primary antibodies targeting γH2AX (2577S, Cell Signaling Technology, Danvers, MA, USA) or H3 (ab1791, Abcam, Cambridge, UK), followed by incubation with a secondary HRP-conjugated goat anti-rabbit IgG (ab6721, Abcam). Levels of γH2AX in each sample were quantified using ImageJ software and normalized to those of histone H3.

### Drug sensitivity assay

Daunorubicin hydrochloride (DNR) was purchased from Merck (Darmstadt, Germany) and dissolved in dimethyl sulfoxide (DMSO). To determine the optimal concentration of DNR for the drug sensitivity assay, K562 cells were seeded at 2 × 10^4^ cells/ml in 100 µl RPMI-1640 growth medium supplemented with 0–30 µM DNR for 72 h with n = 3 biological replicates per concentration [34]. Cell survival was determined using the XTT Cell Viability Assay Kit (Cat. no. 30007, Biotium, Fremont, CA, USA) according to the manufacturer’s instructions, and the fraction of surviving cells was normalized to that of cells incubated in drug-free growth medium.

To determine the relative drug resistance of each sample, cells were seeded at 2 × 10^4^ cells/ml in 100 µl RPMI-1640 growth medium supplemented with 0 or 1 µM DNR for 72 h with n = 3 biological replicates. The proportion of surviving cells was measured by flow cytometry and normalized to that of cells incubated in drug-free growth medium.

### RNA sequencing

RNA sequencing was performed for K562 cells with sgRNAs targeting the following lncRNAs: non-targeting sgRNA 10010, non-targeting sgRNA 10057, *AC005307.3*, *AP006222.2*, *CHD1-DT*, *LINC00221*, *MIR4435-2HG*, *RP11-109M17.2*, *RP11-307E17.8*, *RP11-706O15.3*, and *SNHG6*. Total RNA was extracted from n = 3–4 biological replicates per sample using the PureLink RNA Mini Kit (Invitrogen, Waltham, MA, USA), and sample quality was evaluated using a TapeStation. RNA sequencing was performed using CEL-Seq2 [35], a multiplexed RNA-Seq approach that can be used for pooled bulk RNA sequencing. Raw reads were processed with the Galaxy web platform, using the public server at usegalaxy.org [36]. Briefly, the raw files were converted to FASTQ format using FASTQ Groomer [37], alignment was performed using HISAT2 [38], and counts were generated using featureCounts [39].

Differential gene expression analysis was performed using the NOISeq package in R (version 2.46.0) [40, 41]. Because the samples were sequenced in two batches, a strong batch effect was observed; we performed batch effect correction using the ‘ARSyNseq’ function in NOISeq (Additional file 1: Fig. S2). After correcting for batch effect, we still observed a high proportion of overlapping differentially expressed genes (DEGs) for each hit versus the controls, indicating that many identified DEGs were likely due to batch effect; therefore, we discarded the control samples and instead analyzed each hit compared to the other eight samples to obtain a more accurate indication of the DEGs in each sample. Strongly DEGs were defined as genes with |Log2 Fold Change|> 0.7 and probability > 0.75. Gene Ontology (GO) analysis was performed for the *SNHG6* KD sample based on DEGs using the Enrichr web platform for GO Biological Process [42–44]. To determine the enrichment of TFs associated with the DEGs, the same genes were analyzed using the ChEA3 web platform [45]. To identify the TFs that bind the *SNHG6* promoter in K562 cells, we downloaded the TF binding data for the [− 300; + 100] bp region surrounding the TSS from the ENCODE 3 Transcription Factor ChIP-seq Peaks track in the UCSC genome browser for the hg19 reference genome [46–51].

### AML patient survival analysis

We conducted a survival analysis on the acute myeloid leukemia (AML) dataset from Oregon Health & Science University (OHSU) [52], available on cBioPortal (https://www.cbioportal.org). The dataset included 905 AML samples, with 654 having complete gene expression profiles. We categorized these samples into three quantiles based on *SNHG6* expression levels: low, moderate, and high. To ensure accuracy in assessing the effect of treatment, we excluded patients who died within the first month after diagnosis; this was done because early deaths, particularly in the low *SNHG6* expression group, were likely unrelated to treatment effects. This resulted in a final sample size of 567. Using the ‘survival’ package in R (version 3.5–7), we fitted survival curves to each quantile and calculated the log-rank *p*-value to compare the top and bottom quantiles. We also employed the ‘survminer’ package (version 0.4.9) to generate Kaplan–Meier plots to visually represent the overall survival probability over time across the *SNHG6* expression levels.

### Megakaryocyte differentiation assay

Phorbol 12-myristate 13-acetate (PMA; Merck, Darmstadt, Germany) was dissolved in DMSO. Cells were seeded at 3 × 10^5^ cells/ml in 1 ml RPMI-1640 growth medium supplemented with 0.2 nM PMA for 72 h, with fresh growth medium replaced daily. Megakaryocyte differentiation was determined based on CD41/CD61 membrane protein levels [53]. The fraction of CD41/CD61-positive cells was determined after incubation for 0–72 h by immunofluorescent staining with a PE-conjugated anti-human CD41/CD61 primary antibody (Cat. no. 359805, BioLegend, San Diego, CA, USA) using flow cytometric analysis.

### Erythrocyte differentiation assay

Hemin (Merck, Darmstadt, Germany) was dissolved in DMSO. Cells were seeded at 3 × 10^5^ cells/ml in 1 ml RPMI-1640 growth medium supplemented with 30 µM hemin for 72 h, with fresh growth medium replaced daily. Erythrocyte differentiation was determined based on glycophorin A (GPA) membrane protein levels [54]. The fraction of GPA-positive cells was determined after incubation for 0–72 h by immunofluorescent staining with a PE-conjugated anti-human GPA primary antibody (Cat. no. 349105, BioLegend, San Diego, CA, USA) using flow cytometric analysis.

### Animal experiments

Eight-week-old female Hsd:Athymic nude-*Foxn1*^*nu*^ mice were randomly divided into two groups and injected subcutaneously into the right flank with 1 × 10^6^ K562 cells—with either a non-targeting sgRNA control or *SNHG6*-KD—in 100 µl sterile PBS and 100 µl Corning Matrigel Basement Membrane Matrix (Corning Inc., Corning, NY, USA). Tumor volume was measured every other day starting at day 10 post tumor cell implantation using a vernier caliper and calculated according to the formula (length × width^2^)/2. Mice were sacrificed 20 days after implantation, and the tumors were excised and weighed.

### Statistical analysis

Statistical analyses were performed using the rstatix package in R (version 0.7.2). Values are given as the mean ± SD unless otherwise stated. Significance was evaluated using a two-tailed Student’s *t*-test with Benjamini–Hochberg correction unless otherwise stated. A value of *p* < 0.05 was considered statistically significant.

## Results

### An XGBoost classifier to uncover the function of lncRNAs in cell growth

The human genome encodes more than 15,000 lncRNAs, which are a highly diverse group of genes. While some lncRNAs encode their function in their sequence, others act as DNA regulatory elements, such as enhancers [55]. Furthermore, it remains unclear how many lncRNAs are functional compared to those that are expressed as transcriptional noise. To better understand the biology of lncRNAs, it is necessary to classify them according to their function.

**Fig. 1:**
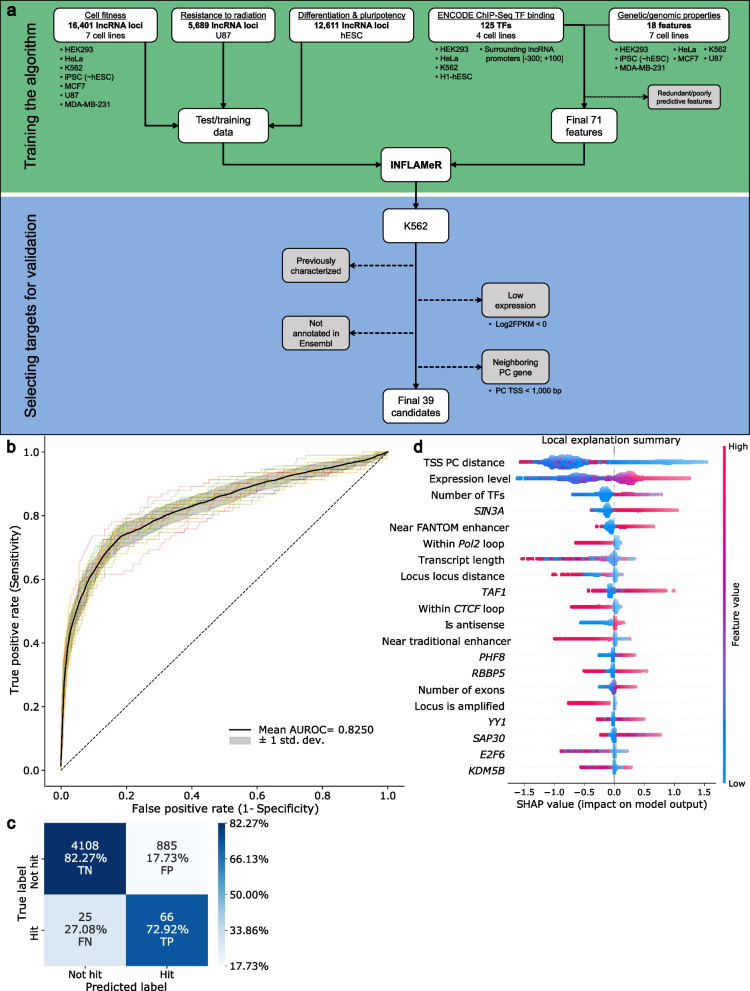
Building a machine learning algorithm for the accurate prediction of novel functional lncRNAs. **a** Workflow for designing the INFLAMeR (Identifying Novel Functional LncRNAs with Advanced Machine learning Resources) algorithm and selecting targets. Upper: The algorithm was trained on previous high-throughput pooled CRISPR interference (CRISPRi) screening data from three previous high-throughput CRISPRi screens [24–26]; the algorithm was computed based on a total of 71 features comprising ENCODE ChIP-Seq transcription factor (TF) binding data for the regions surrounding lncRNA promoters [49–51] and genomic features. Lower: targets predicted to be functional by INFLAMeR in the K562 cell line (INFLAMeR score > 0.5) were selected for validation after excluding lncRNAs that were functionally characterized in previous studies, those with low expression, those not annotated in the Ensembl database, and those neighboring a protein-coding (PC) gene; thirty-nine lncRNAs were selected for validation. **b** INFLAMeR was built using an XGBoost classifier and its performance was calculated using a receiver operating characteristic (ROC) curve. Black ROC curve shows the mean classifier performance on the test set using three randomized seeds (red, green, and yellow curves). **c** Confusion matrix based on the test set. Percentages in the confusion matrix are row-normalized. **d** Local explanation summary of the impact of the top twenty features on the INFLAMeR score. Each dot represents one lncRNA. Red indicates a higher feature value (e.g., larger transcription start site (TSS) protein-coding (PC) distance or higher expression), and blue indicates a lower feature value (e.g., smaller TSS PC distance or lower expression). Higher (red) feature values with a positive SHAP value indicate a positive correlation, and lower (blue) feature values with a positive SHAP value indicate a negative correlation

Over the last several years, ML has been exploited to identify complex relationships between biological properties and predict functionality in various contexts [56]. Herein we developed a binary classification model to identify new functional lncRNAs based upon genetic features, which were trained using known functional lncRNAs (Fig. 1a, upper panel).

To create the ML algorithm, we gathered 143 genetic variables representing different features of lncRNAs (Additional file 1: Table S5). These features included eighteen categorical variables related to lncRNAs and their surrounding genetic regions, such as the transcript length, number of exons, proximity to enhancers, and proximity to PCGs. While these features are important, most do not take into account the cell type-specific functions of lncRNAs. Since most lncRNAs exhibit cell type-specific functionality [1, 5], we also included features pertaining to regulation and expression, namely TF binding data from the ENCODE project for lncRNA promoters in four different cell lines for more than 100 TFs [49–51]. We then trained the ML algorithm using a set of functionally validated lncRNAs. Although many human lncRNAs have been demonstrated to be functional in various contexts, such as cell proliferation [24, 57], differentiation [26, 58], and disease [20, 59, 60], these lncRNAs have often been studied in different cell types and under varying experimental conditions, resulting in scattered data.

In recent years, high throughput reverse genetic screens have been increasingly utilized to explore the functions of numerous lncRNAs [61]. These screens, which involve pooled perturbations, offer a major advantage as they allow thousands of genes to be tested in parallel under identical experimental conditions. The resulting scores generated by these screens are statistically based and enable the classification of genes as either hits (functional) or non-hits. While various perturbation methods have been employed to identify functional lncRNAs, CRISPRi has emerged as the most applied technique across multiple cell types and biological contexts [1].

To train our algorithm, we incorporated data from three genetic screens [24–26] that targeted 16,401 lncRNAs across seven cell lines. However, these data are imbalanced since only 9% (n = 1451) of the lncRNAs were identified as functional. Moreover, perturbation screening of lncRNAs is subject to high false-negative rates due to the inconsistent efficiency of CRISPRi [62] and poor annotation of lncRNA TSSs. This dataset imbalance may introduce a bias into the training algorithm, resulting in diminished predictive value.

To develop the ML model, we experimented with three different cost-sensitive classifiers: logistic regression, balanced random forest, and extreme gradient boosting (XGBoost). We assessed the performance of the model using a range of metrics, including AUROC, sensitivity, specificity, F1-score, precision, and Brier score (Additional file 1: Table S6). Our results show that the mean AUROC for logistic regression was 0.778, which was lower than that for both XGBoost and balanced random forest (mean AUROC values: 0.8236 and 0.8335, respectively; Additional file 1: Fig. S1c).

To determine whether XGBoost or balanced random forest would be better suited for our desired balance of sensitivity and specificity, we determined the percentages of true positive and true negative results for each method (Additional file 1: Fig. S1d). XGBoost had a somewhat higher specificity percentage than balanced random forest (82.24% vs. 80.84%). On average, both XGBoost and balanced random forest produced a similar number of true positive cases (66 and 69 cases, respectively). Additionally, XGBoost possessed two important advantages over balanced random forest. Firstly, in terms of metrics, XGBoost had higher F1-score and precision outcomes (0.1264 and 0.0693, respectively) than balanced random forest (0.1240 and 0.0675, respectively). Secondly, XGBoost had a shorter training time than balanced random forest, which enabled us to experiment and explore more training settings. Therefore, we selected XGBoost for our ML model.

To compensate for the class imbalance problem in our dataset, we tried two different strategies. Initially, we applied random under-sampling of non-hits with and without replacement as preprocessing before training the XGBoost model. We experimented with different sampling strategies to find the optimal ratio of non-hits to hits. The sampling strategies were as follows: 3%, 4%, 5%, 10%, 20%, 30%, 40%, and 50% without and with replacement (Additional file 1: Fig. S1e and f, respectively). We evaluated the performance of each strategy using various metrics such as accuracy, precision, recall, F1-score, and AUROC. We obtained the best performance using the 50% sampling strategy (1822 non-hits and 911 hits), both without and with replacement (Additional file 1: Tables S7 and S8, respectively). We next explored a cost-sensitive approach that assigns different weights to the classes based on their proportion within the dataset, where the algorithm was adjusted to favor the detection of the minority class. This required a modification of the optimization function in the training step of the learning algorithm. For this approach, we implemented several different modifications of the *scale_position_weight* and *class_weight* values. First, we ran the algorithm without a cost value; this means the majority and minority class had the same weight. Second, we applied the default scale position weight, defined as sum(majority class)/sum(minority class). Finally, we applied a *scale_position_weight*/*class_weight* of 100 and 1000. A value of 100 showed the best result, and was used to compare the cost-sensitive XGBoost model with the under-sampled XGBoost models. We found that the cost-sensitive model exhibited superior performance compared to under-sampling with 50% in terms of the AUROC and sensitivity values. Thus, cost-sensitive XGBoost was selected as our ML model.

We used Shapley (SHAP) values [29] to assess the importance of each feature in our XGBoost model for predicting lncRNA function. SHAP values are a useful metric for understanding how a ML model learns from the input features from a global and individualized perspective. SHAP values have several distinct advantages over other explainability approaches such as the gain and split decision tree methods. Firstly, the SHAP approach is model agnostic, meaning the same framework can be used for many different models in addition to XGBoost. More importantly, SHAP values explain the output of a function *f(x)* as a sum of the effects (*φ*) of each feature being introduced into a conditional expectation. Additionally, for nonlinear functions, the order in which features are introduced affects the output. SHAP values are given as the average of all possible orderings, whereas the gain and split methods are inconsistent because they only consider a single ordering [63]. Out of the 143 features, 30 had no predictive value (SHAP ≈ 0) and were hence excluded from further analysis. Although combining multiple features can enhance the predictive power of the model, it can also introduce high-dimensional redundancy and increase the training time and performance bias [64, 65]. Therefore, we applied an RFE method based on SHAP values to select the optimal subset of features that balanced sensitivity and specificity on the test set. Based on RFE, the best performance was obtained with 71 features (Additional file 1: Fig. S1g).

We then evaluated our cost-sensitive XGBoost model with RFE using a triple-repeated tenfold cross-validation with stratified sampling. The hyper-parameters of our gradient-boosted tree classifier were as follows: initial guess of 0.5, gamma of 1.0, gain as importance, learning rate of 0.05, residual-trees with 5 depth levels and 28 leaves (Additional file 1: Fig. S1b), 100 residual-trees, random seed of 0, regularization of lambda of 5.0, and a cost ratio of 100 for misclassifying a hit versus a non-hit.

Using 71 features, the cost-sensitive XGBoost method showed better performance than previous methods. In fact, all metrics were higher for the 71-feature model compared to the 143-feature model; sensitivity and AUROC were increased by 0.05 and 0.002, respectively (Additional file 1: Table S9). We also compared the final XGBoost model to our previous approach using balanced random forest. The XGBoost model with RFE displayed higher mean values of specificity, F1-score, and precision (0.8227 vs. 0.8084, 0.1275 vs. 0.1240, 0.0698 vs. 0.0675, respectively) than the balanced random forest model (Additional file 1: Table S9). Although it did not exhibit the highest score in all metrics, the most important metrics for our analysis—namely the true positive and true negative values, and their balance—were the highest using this strategy, while the rest of the metrics were supplemental.

To estimate the probability of hits among all lncRNAs, we applied our trained model to classify the 50,847 transcripts in our dataset. The INFLAMeR score was given as a value between zero and one, defined as the probability of the transcript to be functional. Transcripts with a score greater than 0.5 were considered to be predicted hits for our analysis. To evaluate the performance of our model, we used a ROC curve and a confusion matrix. Our model achieved a mean AUROC of 0.8250 ± 0.01 (with minimum and maximum values of 0.78 and 0.87, respectively, Fig. 1b; see Additional file 1: Table S10 for all AUROC values), with a true positive rate of 72.92% and a true negative rate of 82.27% (Fig. 1c). Additionally, lncRNAs previously identified as hits tended to have a higher INFLAMeR score (Additional file 6: Table S11), consistent with the high true positive rate.

Statistical analysis of the impact of individual features on the score confirmed the importance of lncRNA-PCG distance (Fig. 1d). Interestingly, the impact of the distance on the prediction score was not linear; rather, we observed a decrease in the score in a stepwise manner, with the SHAP values sharply decreasing around 1000 and 2000 bases from the nearest PCG. However, the distance between promoters did not further affect the score beyond 2000 bases (Additional file 1: Fig. S1h). Further important features were those affecting transcription, such as transcription level (defined as log FPKM), the number of TFs binding to the promoter, and the binding of specific TFs including SIN3A (Fig. 1d). For example, the score of the lncRNA *SNHG6* was decreased owing to its distance from a PCG and proximity to a traditional enhancer; however, it was increased based on its high expression and SIN3A binding at its promoter (Additional file 1: Fig. S1i).

Interestingly, although transcription was tightly correlated with lncRNA functionality (Wilcoxon test, *p* = 4.20 × 10^–222^), the presence of general TFs such as TAF1 and TAF7 did not contribute to the prediction model. These findings suggest that functional lncRNAs may be regulated by a subset of specialized factors.

Our findings indicate that lncRNAs have distinctive features that are closely linked to their functionality. These attributes can be influenced by various factors, such as their genomic location (for example, their proximity to PCGs) and by cell type-specific mechanisms that are intricately regulated by TFs. By identifying these characteristics, one can gain a deeper understanding of the complex roles that lncRNAs may play in regulating genes and cellular processes.

### Validation of INFLAMeR’s predictions and identification of functional lncRNAs

To assess the accuracy of INFLAMeR, we selected a subset of lncRNAs for CRISPRi-mediated knockdown (KD) in K562 cells based on their INFLAMeR score and according to several cutoff parameters (Fig. 1a, lower panel). We excluded the following lncRNAs for validation: those identified in previous screens to be functional in K562 cells; those with low expression (Log2 FPKM < 0) in K562 cells; those which are not annotated in the Ensembl database; and those located within 1 kb of the nearest PCG TSS, as transcription can be initiated from divergent promoters. We included thirty-nine lncRNAs predicted to be functional in K562 cells, with an INFLAMeR score ≥ 0.5. Furthermore, we randomly selected seven lncRNAs that did not meet the INFLAMeR threshold for functional prediction (INFLAMeR score < 0.4) to validate their non-functionality using the same cutoff parameters mentioned above. This was done in order to exclude the possibility that the selected lncRNAs were functional by chance.

Predicting CRISPRi-mediated KD efficiency can be challenging, especially for lncRNAs with multiple splice variants. To enhance KD efficiency, we pooled sgRNAs for each target, with each sample containing two sgRNAs targeting the same lncRNA [66]. We confirmed efficient KD, defined here as relative expression ≤ 0.5 by qPCR, for all the targeted lncRNAs (Additional file 1: Fig. S3a).

**Fig. 2:**
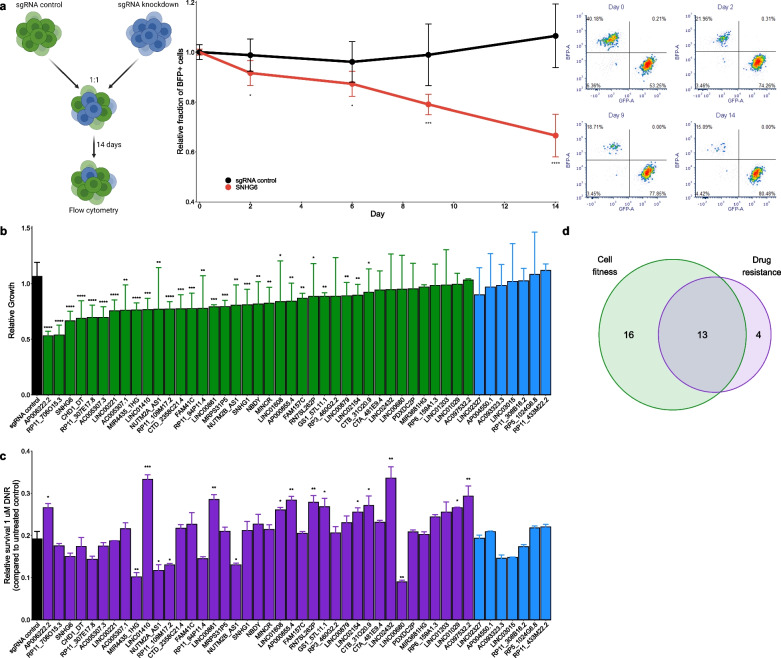
Most lncRNAs predicted to be functional showed a functional effect upon knockdown (KD). **a** Two-color competitive cell growth (CCG) assay. Left: K562 cells stably transduced with sgRNAs targeting a given lncRNA (or non-targeting sgRNA control)—including a blue fluorescent protein (BFP) marker gene—were mixed at a 1:1 ratio with cells transduced with sgRNA control—including a green fluorescent protein (GFP) marker gene—and the fraction of BFP-expressing cells was tracked over 14 days by flow cytometry. The effect of KD on cell proliferation was expressed as the fraction of BFP-expressing cells relative to that at day 0. Middle: a representative example of the change in the relative fraction of BFP-expressing cells throughout the experiment, normalized to day 0. Right: Representative examples of the BFP- and GFP-expressing fractions based on flow cytometry throughout the experiment. **b** The relative growth of BFP-expressing cells after transduction with the sgRNA control (black), thirty-nine lncRNAs predicted to be functional by INFLAMeR (green), and seven lncRNAs predicted to be non-functional (blue) at day 14 of the CCG assay. Error bars represent SD (n = 3 biological replicates). *p < 0.05, **p < 0.01, ***p < 0.001, ****p < 0.0001 vs. sgRNA control using a one-tailed *t*-test. **c** Relative cell survival after 72 h incubation with 1 µM daunorubicin (DNR)—relative to that in untreated cells—for sgRNA control (black), thirty-nine lncRNAs predicted to be functional (purple), and seven lncRNAs predicted to be non-functional (blue). Error bars represent SD (n = 3 biological replicates). *p < 0.05, **p < 0.01, ***p < 0.001 vs. sgRNA control with Bonferroni correction. (**d**) Of the thirty-nine predicted lncRNAs, many of those with an effect on cell proliferation (green) also affected resistance to DNR (purple) upon KD

To determine whether the validated lncRNAs affected cell proliferation upon KD, we conducted a two-color CCG assay for each sample (Fig. 2a); briefly, cells with KD of each lncRNA (or transduced with a non-targeting sgRNA) expressing BFP were mixed at a 1:1 ratio with cells transduced with a non-targeting sgRNA expressing GFP, and the relative proportion of BFP-positive cells was measured over 14 days by flow cytometry to determine the impact of KD on cell proliferation. We observed a significant reduction in the proportion of BFP-positive cells (*p* < 0.05) for 74% of the samples predicted to be functional (n = 29/39), while none of the seven lncRNAs predicted to be non-functional had a significant impact on cell proliferation (Fig. 2b).

Importantly, we did not observe any enhancement in cell proliferation following the knockdown of lncRNAs. This outcome aligns with the intrinsic characteristics of our cell model, derived from leukemia, where cells are already optimized for maximal proliferation under favorable growth conditions. Hence, the knockdown of lncRNAs is more likely to manifest as a reduction rather than an enhancement of proliferation rates.

Since lncRNAs often function in *cis*, we investigated whether the validated genes were adjacent to PCGs considered essential based on dependency scores obtained using the DepMap portal, which characterizes essential genes based on gene perturbation across more than 1000 cancer cell lines [67]. We found no correlation between the INFLAMeR score of the thirty-nine predicted lncRNAs and the essentiality of their neighboring PCGs, further supporting the independent function of the lncRNAs (Additional file 1: Fig. S4).

The observed effect on cell fitness may be the cumulative result of alterations in cell cycle and cell viability. Therefore, we further analyzed changes in cell cycle and apoptosis for eleven lncRNAs that showed the most dramatic effect on cell proliferation. We observed significant changes in the cell cycle for nine lncRNAs (Additional file 1: Fig. S5a). In agreement with this finding, we also observed an increase in the fraction of apoptotic cells in six of these samples (Additional file 1: Fig. S5b), as well as increased basal DNA damage in nine samples, as determined based on increased levels of the *bona fide* DNA damage marker γH2AX (Additional file 1: Fig. S5c).

However, CRISPRi may result in off-target effects owing to the genomic positioning of the target gene or low-specificity interactions of the sgRNA caused by sequence similarity. Therefore, to validate the on-target specificity of our approach, we performed TSS deletion using Cas9 with dual sgRNAs. To this end, we tested three pairs of sgRNAs flanking the TSS for each of the top ten lncRNAs. For most genes (n = 8/10), we achieved a significant reduction in lncRNA expression from one sgRNA pair (Additional file 1: Fig. S3b). Importantly, most of these cells showed the same impact on cell cycle regulation (Additional file 1: Fig. S6a) and apoptosis (Additional file 1: Fig. S6b) as those observed after CRISPRi, indicating the gene-specific effect of our findings.

Studying cell proliferation under optimal growth conditions may overlook lncRNAs that function in response to stress cues. Therefore, we incubated each sample with DNR, an anthracycline chemotherapeutic agent commonly used to treat leukemias [34]; DNR exerts its pharmacologic activity as a DNA topoisomerase II inhibitor as well as a DNA intercalator. After confirming the optimal concentration of DNR for the growth inhibition assay based on control cells and ten lncRNAs (Additional file 1: Fig. S7; see methods), each sample was incubated with 1 µM DNR for 72 h to determine whether these lncRNAs affected DNR activity. Of the thirty-nine hits, 44% (n = 17/39) exhibited a significant effect on DNR activity (Fig. 2c), while the seven non-hits did not affect DNR resistance. Of the seventeen lncRNAs affecting DNR resistance, thirteen also showed a significant reduction in cell proliferation from the CCG assay (Fig. 2d).

Thus, 85% (n = 33/39) of the lncRNAs predicted by INFLAMeR to be functional were experimentally validated to have a function in either cell proliferation or anticancer drug resistance. Importantly, most of these lncRNAs have never been functionally characterized until now, and none of them has a known function in K562 cells.

Collectively, these findings demonstrate the utility of INFLAMeR in predicting the functionality of lncRNAs.

### Characterization of top lncRNAs affecting cell proliferation

**Fig. 3:**
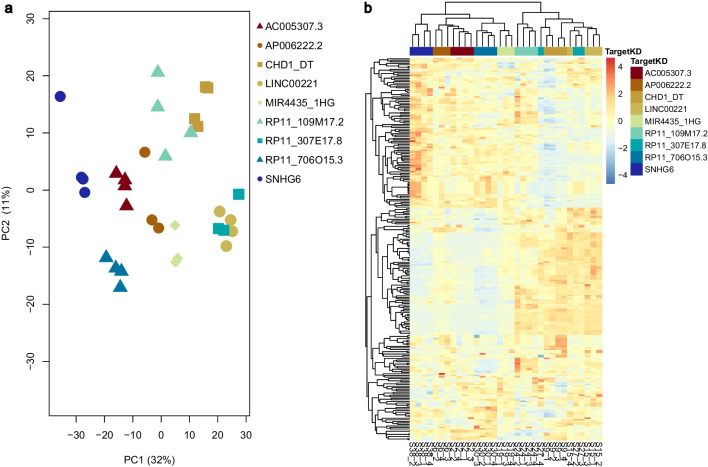
Transcriptome analysis for nine validated lncRNAs. **a** Principal component analysis was performed for the top variable genes in each sample to confirm that each lncRNA affected a distinct group of genes upon knockdown (KD). **b** Differential gene expression analysis indicated that most of the lncRNAs affected a unique subset of genes upon KD

To further elucidate the functional mechanisms of nine lncRNAs significantly influencing cell proliferation upon KD, we performed transcriptome analyses. Principal component analysis (PCA) displayed distinct clustering for each lncRNA (Fig. 3a). Notably, for most lncRNAs, we identified a relatively modest number of highly differentially expressed genes (DEGs), ranging from 70 to 300 DEGs—characterized as having a |Log2 Fold Change|> 0.7 and a probability > 0.75. Additionally, analyzing the most variable DEGs for each lncRNA revealed unique gene expression patterns in the samples (Fig. 3b, Additional file 1: Fig. S8). This suggests that although KD of each lncRNA hindered cell proliferation, it likely occurred through distinct mechanisms. We also noted that for all nine lncRNAs under study, gene expression was impacted across the genome (Additional file 1: Fig. S9).

LncRNAs can affect local transcription (*cis*) or operate via distal interactions (*trans*). To gain a deeper understanding of their functions, we overexpressed ten of these lncRNAs through pseudo-lentiviral transduction (Additional file 1: Fig. S3c). Overexpression of these lncRNAs led to a partial or complete rescue of cell cycle regulation and apoptosis (Additional file 1: Fig. S10a and b, respectively). Among the genes investigated, *SNHG6* emerged as an especially noteworthy candidate.

### *SNHG6* regulates hematopoiesis in K562 cells

**Fig. 4:**
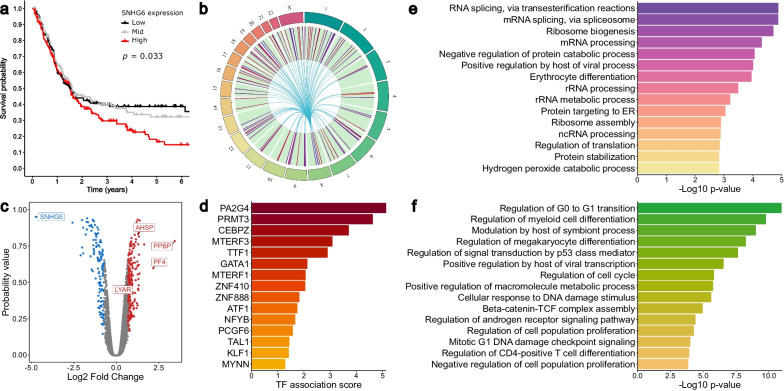
Small nucleolar host gene 6 (*SNHG6*) acts as a regulator of hematopoiesis. **a** High *SNHG6* expression is associated with reduced overall survival in acute myeloid leukemia. **b** *SNHG6* knockdown (KD) affects the expression of genes across the genome, implying a *trans* regulatory function. Blue represents downregulation, red represents upregulation. **c** *SNHG6* KD led to the upregulation of the platelet-associated genes *PPBP* and *PF4*, and the erythrocyte-associated genes *AHSP* and *LYAR*. **d** Transcription factors (TFs) strongly associated with the differentially expressed genes (DEGs) upon *SNHG6* KD include hematopoiesis-associated TFs such as CEBPZ, GATA1, TAL1, and KLF1. **e** Gene Ontology (GO) analysis of the DEGs indicates a strong correlation with erythrocyte differentiation. **f** GO analysis of the TFs known to bind *SNHG6* in K562 cells shows a strong association with myeloid differentiation- and hematopoiesis-related pathways

*SNHG6* is a lncRNA with a known impact in several cancer types [68–74], and is significantly associated with a poor prognosis in acute myeloid leukemia (Fig. 4a).

**Fig. 5:**
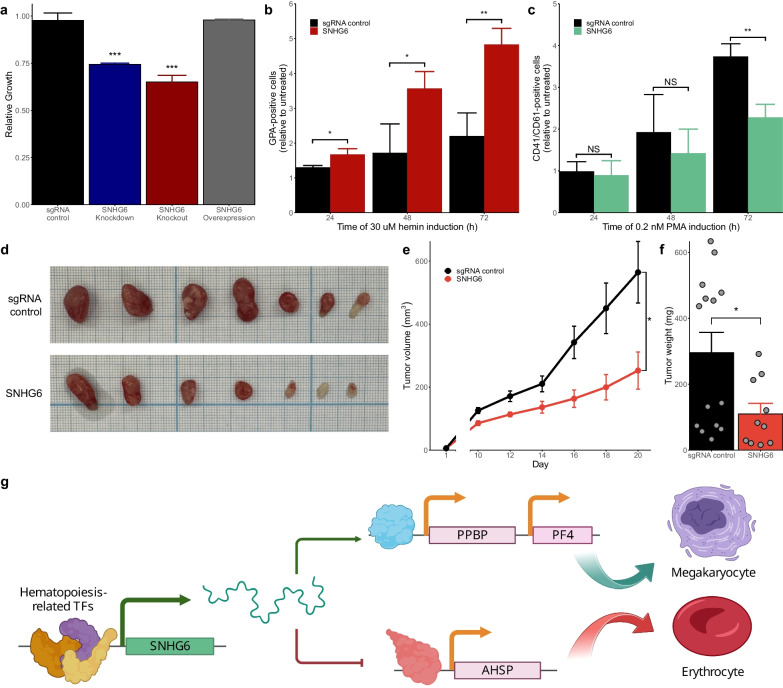
*SNHG6* KD promotes erythrocyte differentiation and prevents megakaryocyte differentiation in K562 cells. **a** Cell proliferation was significantly reduced by both knockdown (KD) and knockout, while overexpression in KD samples restored proliferation rates. **b** GPA levels were normalized to those in untreated cells in each sample to quantify the degree of erythrocyte differentiation. *SNHG6* KD promoted erythrocyte differentiation. Error bars represent SD (n = 3 biological replicates). *p < 0.05, **p < 0.01 vs. sgRNA control. **c** CD41/CD61 levels were normalized to basal levels in each sample to quantify the degree of megakaryocyte differentiation. *SNHG6* KD significantly reduced megakaryocyte differentiation after 72 h. Error bars represent SD (n = 3 biological replicates). **p < 0.01 vs. sgRNA control. **d**–**f** *SNHG6* KD led to reduced tumor volume (**e**) and weight (**f**) in a xenograft mouse model. **d** Representative tumors. Error bars represent SEM. *p < 0.05 vs. sgRNA control by one-way ANOVA (n ≥ 11 from two independent experiments). **g** Proposed model for the mechanism of function of *SNHG6*

The KD of *SNHG6* led to the differential expression of 304 genes, including 126 protein-coding genes and 103 lncRNAs. The identified DEGs were widespread across the entire genome (Fig. 4b), suggesting a *trans* regulatory role for *SNHG6*. This was consistent with the cytoplasmic localization of *SNHG6* in K562 cells (Additional file 1: Fig. S11), and the observed phenotypic rescue caused by overexpression (Fig. 5a and Additional file 1: Fig. S10).

Interestingly, *SNHG6* KD led to the upregulation of genes involved in myeloid differentiation to both erythrocyte and megakaryocyte lineages. Following *SNHG6* KD, the most strongly up-regulated genes included the erythrocyte-associated α-hemoglobin stimulating protein (*AHSP*) and Ly1-antibody reactive (*LYAR*); and the megakaryocyte-associated pro-platelet basic protein (*PPBP*) and platelet factor 4 (*PF4*) (Fig. 4c). Using ChEA3, a tool for identifying the TFs most strongly associated with the DEGs [45], we observed a strong correlation with several TFs associated with hematopoietic differentiation, including CEBPZ [75], KLF1, TAL1, and GATA1 [76] (Fig. 4d). Consistent with the erythroleukemia origin of K562 cells, GO analysis using Enrichr [42–44] showed strong enrichment for erythrocyte differentiation (Fig. 4e).

We also performed GO analysis of the TFs targeting the *SNHG6* promoter. Expectedly, the most strongly enriched pathways were associated with transcription (Additional file 1: Fig. S12). Surprisingly, however, there were several highly enriched pathways relating to hematopoietic and myeloid differentiation (Fig. 4f). These findings are in line with recent reports showing that *SNHG6* plays a role in myeloid cell differentiation in mice, and computational predictions suggesting a role in leukemia progression and patient prognosis [70, 77].

### *SNHG6* regulates hematopoietic differentiation in K562 cells

To confirm that the observed cell proliferation phenotype was caused by the KD of *SNHG6*, we performed a CCG assay on samples after *SNHG6* KO and overexpression. Deletion of the *SNHG6* TSS caused a similar reduction in cell proliferation to that observed upon KD, and the growth phenotype was rescued by *SNHG6* overexpression (Fig. 5a).

Next, to corroborate these findings, we assessed the differentiation potential of *SNHG6*-KD cells.

To stimulate erythrocyte differentiation, *SNHG6*-KD and control cells were incubated with hemin, and erythrocyte status was confirmed based on the levels of the surface marker GPA after 24, 48, and 72 h (Additional file 1: Fig. S13a). Starting at 24 h, we found a significant increase in GPA levels in *SNHG6*-KD cells compared to that in sgRNA control cells (Fig. 5b), indicating an increased affinity for erythrocyte differentiation.

To stimulate megakaryocyte differentiation, cells were incubated with PMA, and megakaryocyte status was confirmed based on the levels of the CD41/CD61 surface marker after 24, 48, and 72 h. We found that basal CD41/CD61 levels were decreased in *SNHG6*-KD cells compared to those in control cells (Additional file 1: Fig. S13b). After 72 h, CD41/CD61 levels were significantly lower in *SNHG6*-KD cells than in sgRNA control cells (Fig. 5c), indicating a decreased stimulation of megakaryocyte differentiation.

Finally, we investigated the impact of *SNHG6* KD on K562 proliferation in vivo. To this end, we established a mouse leukemia xenograft model by injecting mice with K562 cells containing our non-targeting sgRNA control or *SNHG6*-KD (n ≥ 11 mice per group; Fig. 5d). Tumor volume was measured starting at 10 days after K562 cell implantation, and the tumors were significantly smaller in the *SNHG6*-KD group compared to the control throughout the experiment (Fig. 5e). After 20 days, the mice were sacrificed and tumors were excised and weighed. Consistent with the observed effect on tumor volume, the overall weight of *SNHG6*-KD tumors was also significantly lower than that in control tumors (Fig. 5f). These findings confirm the role of SNHG6 in K562 cell proliferation in vitro and in vivo.

Together, our findings show that *SNHG6* KD promotes erythrocyte differentiation and inhibits megakaryocyte differentiation in K562 cells, indicating that *SNHG6* acts as a regulator of hematopoietic differentiation (Fig. 5f).

## Discussion

LncRNAs represent a large and heterogenous group of genes. To enhance our understanding of their biological roles, there is a need to identify subgroups and cluster them accordingly. To this end, the comprehensive characterization of functional lncRNAs is critical. However, this task has proved to be challenging not only due to their diverse functional mechanisms, but also due to their low expression and cell type-specific functions [1, 5].

Here, we present INFLAMeR, an ML-based tool for the classification and prediction of functional lncRNAs. INFLAMeR uses both constant and variable genomic features that allow for the prediction of functional lncRNAs in a cell type-specific manner. The variability in lncRNA expression between cell types and cellular conditions indicates that their expression is tightly regulated. Therefore, INFLAMeR was built based on a large set of TFs occupying lncRNA promoters. Indeed, our current analysis of the contribution of different features based on SHAP values indicated that TFs are important contributors for lncRNA classification. Interestingly, functional prediction was correlated with an increased number of TFs; this was the third most strongly contributing feature. This may indicate that the combinatorial effect of multiple factors is critical for their function, rather than the role of a specific TF.

Surprisingly, while the distance between lncRNAs and the closest PCG was the most strongly contributing feature for classification, we did not find a correlation between lncRNAs affecting cell proliferation and the dependency scores of the PCGs neighboring each lncRNA (Additional file 1: Fig. S4). Furthermore, although *cis* regulation is well documented as a mechanism of function for many lncRNAs [78], our transcriptomic analysis following KD of validated lncRNAs did not identify significant changes in the expression of most neighboring PCGs. This suggests that INFLAMeR enriched for *trans* regulating lncRNAs.

Our use of INFLAMeR reveals a large number of false negative results in pooled CRISPRi perturbation screens. We can point out two contributing reasons. Firstly, previous CRISPRi screens transduced only one sgRNA in each cell, which often results in low KD efficiency. Using two sgRNAs targeting the same gene, we achieved a much stronger KD, which allowed us to reveal the function of the targeted lncRNAs. Secondly, in most cases, the overall effect of lncRNA KD on cell proliferation or anticancer drug resistance is relatively mild, as can be expected for regulatory genes. This may result in a low signal-to-noise ratio in a pooled screen. Hence, the improvement of KD efficiency together with post-screening analysis by algorithms such as INFLAMeR may increase the selectivity of perturbation screens and reveal many more functional lncRNAs.

Importantly, intensive validation showed clear functionality for 85% of lncRNAs displaying a high INFLAMeR score, but no functionality for genes with a low score. This confirms the high accuracy of our predictions. Functional validation of several top performing hits also revealed that the observed effect on tumor cell proliferation was due to dysregulated cell cycle progression, apoptosis, and DNA damage repair. To rule out the possibility of off-target effects, we performed TSS deletion for selected hits and observed a similar phenotype. Additionally, the overexpression of a subset of our hits successfully reversed the phenotype observed by KD. A reversal of the phenotype was not seen for all of our overexpressed hits; this is likely because the function of these lncRNAs is closely related to their location on the genome, either as *cis*-acting lncRNAs or as regulatory elements at the DNA level. Further transcriptomics characterization of the top validated lncRNAs showed that while the number of DEGs was relatively small, each lncRNA affected a different subset of genes. Interestingly, in several cases, lncRNA KD resulted in the coordinated expression of genes in the same locus. However, in the vast majority of cases, the target genes were not neighboring the lncRNA, indicating the *trans*-genomic changes induced by these lncRNAs.

Our analysis identified *SNHG6* as a functionally important lncRNA. We found that *SNHG6* KD attenuated tumor cell proliferation both in vitro and in vivo, but did not affect the response to a *bona fide* cytotoxic agent. These findings are consistent with our transcriptome analysis, which revealed that *SNHG6* regulates hematopoietic differentiation. Both erythrocyte-specific genes such as hemoglobin subunits, as well as platelet-specific genes such as *PPBP* and *PF4*, were differentially expressed following *SNHG6* KD. This KD further caused the erythroleukemic cell line K562 to be susceptible to erythrocyte differentiation, but resistant to megakaryocyte differentiation. Further studies are warranted to determine whether the observed function of *SNHG6* was caused by the lncRNA itself, or by its contained snoRNA. Additionally, there is recent evidence that *SNHG6* encodes for a small peptide that may be functional [79].

## Conclusion

Overall, we show that INFLAMeR can be trained to readily identify functional lncRNAs in diverse cells and tissue types, as well as under distinct biological cues and contexts. Furthermore, INFLAMeR can be used to enhance the sensitivity and specificity of large-scale perturbation screens by constructing more efficient sgRNA libraries.

### Supplementary Information

**Additional file 1: Fig. S1.** Building the machine learning algorithm. **a** Process followed for model training. The functional screening based on CRISPRi and the ENCODE Transcription Factor datasets were split into 90% for the training set (adopting a stratified cross-validation) and 10% for the testing set; along with binary labels indicating whether the lncRNA locus is either a hit or not hit. **b** XGBoost first residual-tree. Tree nodes are represented as rounded grey boxes, and squared white boxes are the tree leafs. **c** ROC curves comparing XGBoost, balanced random forest, and logistic regression models. **d** XGBoost (upper) and balanced random forest (lower) confusion matrices. Models were trained on 90% of the data, and ROC curves and confusion matrices show predictive value on the remaining 10%. Percentages from confusion matrices are row-normalized. **e**, **f** Under-sampling PCA. PCA of random under-sampling of the majority class (i.e. not hit) without (**e**) and with (**f**) replacement, plotting the complete dataset (upper-left plot) plus 8 sampling strategies. PCA values based on 130 numeric features showing the removed not hit transcripts. Red dots: hit; grey dots: not hit. **g** Recursive feature elimination. Iteratively, one feature was removed to train a new model, removing the least important. Red and black lines denote the optimal number of features (n = 71) and sensitivity value using 143 features, respectively. **h** SHAP dependence plot for the TSS PC distance feature. Each blue dot denotes a lncRNA. Positive odd values (above dashed line) contribute towards prediction of hits. **i** Detailed explanation for the INFLAMeR score of SNHG6. The final INFLAMeR score of SNHG6 was 0.504. **Fig. S2.** sgRNA control samples show distinct clustering after batch effect correction. After batch effect correction using the NOISeq package, the samples were clustered according to the target KD; however, the sgRNA control samples were distinctly clustered from all lncRNA KD samples. **Fig. S3.** Confirming the change in lncRNA expression after knockdown (KD), knockout (KO), and overexpression by qPCR. **a** KD of thirty-nine lncRNAs predicted to be functional (red) and seven lncRNAs predicted to be non-functional (blue). **b** KO of eight top performing lncRNAs. **c** Confirming overexpression of ten top performing lncRNAs following stable transduction of a lentiviral plasmid containing the lncRNA sequence. Expression is given relative to that in samples transduced with a non-targeting control sgRNA. **Fig. S4.** There was no correlation between INFLAMeR score and the essentiality of neighboring protein-coding (PC) genes. Genes with a dependency score > 1 (red line) are considered essential. Green line represents the linear regression with 95% confidence interval (grey). **Fig. S5.** Knockdown of top performing lncRNAs affects cell cycle, apoptosis, and DNA damage. Knockdown of the indicated lncRNAs caused dysregulation of the cell cycle (**a**), increased apoptosis rates (**b**), and increased rates of DNA damage (**c**), as indicated by increased levels of γH2AX. *p < 0.05, **p < 0.01, ***p < 0.001, ****p < 0.0001 vs. sgRNA control (n = 3). **Fig. S6.** Knockout of top performing lncRNAs affects cell cycle and apoptosis. Functional knockout of the indicated lncRNAs by TSS deletion caused dysregulation of the cell cycle (**a**) and increased apoptosis rates (**b**), replicating the results seen after knockdown (See Fig. S4). *p < 0.05, **p < 0.01, ***p < 0.001, ****p < 0.0001 vs. sgRNA control (n = 3). **Fig. S7.** Determining the IC50 for daunorubicin (DNR) of selected samples. K562 cells from the indicated samples were incubated with the indicated concentrations of DNR for 72 h and their viability was determined using the XTT assay relative to that in untreated cells. Values are given as the mean ± SD for n = 3 biological replicates. The black line in each curve represents the non-targeting sgRNA control sample. * *p *< 0.05, **p < 0.01, ***p < 0.001 vs. sgRNA control. **Fig. S8.** Differentially expressed genes from each sample. KD of the indicated lncRNAs generally led to a higher proportion of downregulated genes (blue) compared to upregulated genes (red). **Fig. S9.** KD of the indicated lncRNAs affected the expression of genes across the genome. Red represents upregulation, blue represents downregulation. **Fig. S10.** Overexpression of top performing lncRNAs rescued cell cycle regulation and apoptosis rates. Rescuing the expression of the indicated lncRNAs after knockdown partially or fully restored cell cycle regulation (**a**) and reduced apoptosis rates (**b**) for most of the samples (See Fig. S4). *p < 0.05, **p < 0.01 vs. sgRNA control (n = 3). **Fig. S11.** SNHG6 subcellular localization. The enrichment of SNHG6 was measured by qPCR in the nuclear/cytoplasmic fractions of K562 cells. MALAT1 and GAPDH were used as nuclear and cytoplasmic controls, respectively. **Fig. S12.** Gene ontology analysis for the transcription factors that bind the promoter of SNHG6 in K562. The top 50 pathways are shown. **Fig. S13**. Measuring the levels of myeloid differentiation markers by flow cytometry. **a** Erythrocyte differentiation was assessed based on glycophorin A (GPA) levels using immunostaining with flow cytometry in cells incubated with 30 µM hemin for 72 h. **b** Megakaryocyte differentiation was measured based on CD41/CD61 levels using immunostaining with flow cytometry in cells incubated with 0.2 nM PMA for 72 h. **Table S5.** The 143 features included in the initial algorithm. **Table S6.** Cost-sensitive model metrics. **Table S7.** Under-sampling strategies without replacement. **Table S8.** Under-sampling strategies with replacement. **Table S9.** Model performance comparison. **Table S10.** Performance of 10-fold cross-validation (CV).

**Additional file 2: Table S1.** Features included in the machine learning algorithm.

**Additional file 3: Table S2.** Primer sequences for qPCR and colony PCR.

**Additional file 4: Table S3.** sgRNA sequences used in this study.

**Additional file 5: Table S4.** Sequences used for lncRNA overexpression.

**Additional file 6: Table S11.** The INFLAMeR score of the genes identified as positive hits in the previous large-scale screen.

## Data Availability

The data used to train the ML models with 143 features is available in Zenodo at: 10.5281/zenodo.10251230. The data after RFE with 71 features is available in Zenodo at: 10.5281/zenodo.8114662. The code used to train and test our INFLAMeR ML model is available in the following GitHub repository: https://github.com/razielar/INFLAMer.

## Abbreviations

lncRNA: Long non-coding RNA
CRISPRi: CRISPR interference
INFLAMeR: Identifying Novel Functional LncRNAs with Advanced Machine Learning Resources
ML: Machine learning
KD: Knockdown
SNHG6: Small Nucleolar RNA Host Gene 6
PPBP: Pro-platelet basic protein
PF4: Platelet factor 4
AHSP: Alpha hemoglobin stimulating protein
LYAR: Ly1-antibody reactive
TF: Transcription factor
PCG: Protein-coding gene
TSS: Transcription start site
ROC: Receiver operating characteristic
AUROC: Area under the receiver operating characteristic curve
RFE: Recursive feature elimination
CCG: Competitive cell growth
BFP: Blue fluorescent protein
GFP: Green fluorescent protein
DNR: Daunorubicin
DEG: Differentially expressed gene
GO: Gene ontology
GPA: Glycophorin A
PMA: Phorbol 12-myristate 13-acetate
DMSO: Dimethyl sulfoxide

## References

[1] Hazan J, Bester AC (2021). CRISPR-based approaches for the high-throughput characterization of long non-coding RNAs. Noncoding RNA.

[2] Mattick JS, Amaral PP, Carninci P, Carpenter S, Chang HY, Chen LL (2023). Long non-coding RNAs: definitions, functions, challenges and recommendations. Nat Rev Mol Cell Biol.

[3] Camilleri-Robles C, Amador R, Klein CC, Guigó R, Corominas M, Ruiz-Romero M (2022). Genomic and functional conservation of lncRNAs: lessons from flies. Mamm Genome.

[4] Zhao Y, Li H, Fang S, Kang Y, Wu W, Hao Y (2016). NONCODE 2016: an informative and valuable data source of long non-coding RNAs. Nucleic Acids Res.

[5] Ulitsky I (2016). Evolution to the rescue: using comparative genomics to understand long non-coding RNAs. Nat Rev Genet.

[6] Gao F, Cai Y, Kapranov P, Xu D (2020). Reverse-genetics studies of lncRNAs-what we have learnt and paths forward. Genome Biol.

[7] Ramilowski JA, Yip CW, Agrawal S, Chang JC, Ciani Y, Kulakovskiy IV (2020). Functional annotation of human long noncoding RNAs via molecular phenotyping. Genome Res.

[8] Nötzold L, Frank L, Gandhi M, Polycarpou-Schwarz M, Groß M, Gunkel M (2017). The long non-coding RNA LINC00152 is essential for cell cycle progression through mitosis in HeLa cells. Sci Rep.

[9] Korkmaz G, Lopes R, Ugalde AP, Nevedomskaya E, Han R, Myacheva K (2016). Functional genetic screens for enhancer elements in the human genome using CRISPR-Cas9. Nat Biotechnol.

[10] Liu Y, Cao Z, Wang Y, Guo Y, Xu P, Yuan P (2018). Genome-wide screening for functional long noncoding RNAs in human cells by Cas9 targeting of splice sites. Nat Biotechnol.

[11] Zhu S, Li W, Liu J, Chen CH, Liao Q, Xu P (2016). Genome-scale deletion screening of human long non-coding RNAs using a paired-guide RNA CRISPR-Cas9 library. Nat Biotechnol.

[12] Tao M, Mu Q, Zhang Y, Xie Z (2020). Construction of a CRISPR-based paired-sgRNA library for chromosomal deletion of long non-coding RNAs. Quant Biol.

[13] Fulco CP, Munschauer M, Anyoha R, Munson G, Grossman SR, Perez EM (2016). Systematic mapping of functional enhancer-promoter connections with CRISPR interference. Science.

[14] Joung J, Engreitz JM, Konermann S, Abudayyeh OO, Verdine VK, Aguet F (2017). Genome-scale activation screen identifies a lncRNA locus regulating a gene neighbourhood. Nature.

[15] Bester AC, Lee JD, Chavez A, Lee YR, Nachmani D, Vora S (2018). An integrated genome-wide CRISPRa approach to functionalize lncRNAs in drug resistance. Cell.

[16] Xu D, Cai Y, Tang L, Han X, Gao F, Cao H (2020). A CRISPR/Cas13-based approach demonstrates biological relevance of vlinc class of long non-coding RNAs in anticancer drug response. Sci Rep.

[17] Carlevaro-Fita J, Lanzós A, Feuerbach L, Hong C, Mas-Ponte D, Pedersen JS (2020). Cancer LncRNA Census reveals evidence for deep functional conservation of long noncoding RNAs in tumorigenesis. Commun Biol.

[18] Kirk JM, Kim SO, Inoue K, Smola MJ, Lee DM, Schertzer MD (2018). Functional classification of long non-coding RNAs by k-mer content. Nat Genet.

[19] Ehsani R, Drabløs F (2018). Measures of co-expression for improved function prediction of long non-coding RNAs. BMC Bioinform.

[20] Pyfrom SC, Luo H, Payton JE (2019). PLAIDOH: a novel method for functional prediction of long non-coding RNAs identifies cancer-specific LncRNA activities. BMC Genom.

[21] Fernández M, Miranda-Saavedra D (2012). Genome-wide enhancer prediction from epigenetic signatures using genetic algorithm-optimized support vector machines. Nucleic Acids Res.

[22] Wen J, Liu Y, Shi Y, Huang H, Deng B, Xiao X (2019). A classification model for lncRNA and mRNA based on k-mers and a convolutional neural network. BMC Bioinform.

[23] Zhang J, Zhang Z, Wang Z, Liu Y, Deng L (2018). Ontological function annotation of long non-coding RNAs through hierarchical multi-label classification. Bioinformatics.

[24] Liu SJ, Horlbeck MA, Cho SW, Birk HS, Malatesta M, He D (2017). CRISPRi-based genome-scale identification of functional long noncoding RNA loci in human cells. Science.

[25] Liu SJ, Malatesta M, Lien BV, Saha P, Thombare SS, Hong SJ (2020). CRISPRi-based radiation modifier screen identifies long non-coding RNA therapeutic targets in glioma. Genome Biol.

[26] Haswell JR, Mattioli K, Gerhardinger C, Maass PG, Foster DJ, Fernandez PP (2021). Genome-Wide CRISPR interference screen identifies long non-coding RNA loci required for differentiation and pluripotency. SSRN Electron J.

[27] Dao LTM, Galindo-Albarrán AO, Castro-Mondragon JA, Andrieu-Soler C, Medina-Rivera A, Souaid C (2017). Genome-wide characterization of mammalian promoters with distal enhancer functions. Nat Genet.

[28] Chen T, Guestrin C. XGBoost: a scalable tree boosting system. In: Proceedings of the ACM SIGKDD International Conference on Knowledge Discovery and Data Mining. New York: ACM; 2016. p. 785–94. 10.1145/2939672.2939785

[29] Shapley LS, Kuhn HW, Tucker AW (1953). A value for n-person games. Contribution to the theory of games.

[30] Pulido-Quetglas C, Aparicio-Prat E, Arnan C, Polidori T, Hermoso T, Palumbo E (2017). Scalable design of paired CRISPR guide RNAs for genomic deletion. PLoS Comput Biol.

[31] Cao XM, Luo XG, Liang JH, Zhang C, Meng XP, Guo DW (2012). Critical selection of internal control genes for quantitative real-time RT-PCR studies in lipopolysaccharide-stimulated human THP-1 and K562 cells. Biochem Biophys Res Commun.

[32] Eekels JJM, Pasternak AO, Schut AM, Geerts D, Jeeninga RE, Berkhout B (2012). A competitive cell growth assay for the detection of subtle effects of gene transduction on cell proliferation. Gene Ther.

[33] Kuo LJ, Yang L-X (2008). Gamma-H2AX—a novel biomarker for DNA double-strand breaks. In Vivo.

[34] Levin M, Stark M, Ofran Y, Assaraf YG (2021). Deciphering molecular mechanisms underlying chemoresistance in relapsed AML patients: towards precision medicine overcoming drug resistance. Cancer Cell Int.

[35] Hashimshony T, Senderovich N, Avital G, Klochendler A, de Leeuw Y, Anavy L (2016). CEL-Seq2: Sensitive highly-multiplexed single-cell RNA-Seq. Genome Biol.

[36] Afgan E, Nekrutenko A, Grüning BA, Blankenberg D, Goecks J, Schatz MC (2022). The Galaxy platform for accessible, reproducible and collaborative biomedical analyses: 2022 update. Nucleic Acids Res.

[37] Blankenberg D, Gordon A, Von Kuster G, Coraor N, Taylor J, Nekrutenko A (2010). Manipulation of FASTQ data with galaxy. Bioinformatics.

[38] Kim D, Langmead B, Salzberg SL (2015). HISAT: a fast spliced aligner with low memory requirements. Nat Methods.

[39] Liao Y, Smyth GK, Shi W (2014). FeatureCounts: an efficient general purpose program for assigning sequence reads to genomic features. Bioinformatics.

[40] Tarazona S, García-Alcalde F, Dopazo J, Ferrer A, Conesa A (2011). Differential expression in RNA-seq: a matter of depth. Genome Res.

[41] Tarazona S, Furió-Tarí P, Turrà D, Di Pietro A, Nueda MJ, Ferrer A (2015). Data quality aware analysis of differential expression in RNA-seq with NOISeq R/Bioc package. Nucleic Acids Res.

[42] Xie Z, Bailey A, Kuleshov MV, Clarke DJB, Evangelista JE, Jenkins SL (2021). Gene set knowledge discovery with Enrichr. Curr Protoc.

[43] Chen EY, Tan CM, Kou Y, Duan Q, Wang Z, Meirelles GV (2013). Enrichr: interactive and collaborative HTML5 gene list enrichment analysis tool. BMC Bioinform.

[44] Kuleshov MV, Jones MR, Rouillard AD, Fernandez NF, Duan Q, Wang Z (2016). Enrichr: a comprehensive gene set enrichment analysis web server 2016 update. Nucleic Acids Res.

[45] Keenan AB, Torre D, Lachmann A, Leong AK, Wojciechowicz ML, Utti V (2019). ChEA3: transcription factor enrichment analysis by orthogonal omics integration. Nucleic Acids Res.

[46] Gerstein MB, Kundaje A, Hariharan M, Landt SG, Yan KK, Cheng C (2012). Architecture of the human regulatory network derived from ENCODE data. Nature.

[47] Wang J, Zhuang J, Iyer S, Lin XY, Whitfield TW, Greven MC (2012). Sequence features and chromatin structure around the genomic regions bound by 119 human transcription factors. Genome Res.

[48] Wang J, Zhuang J, Iyer S, Lin XY, Greven MC, Kim BH (2013). Factorbook.org: A Wiki-based database for transcription factor-binding data generated by the ENCODE consortium. Nucleic Acids Res.

[49] Sloan CA, Chan ET, Davidson JM, Malladi VS, Strattan JS, Hitz BC (2016). ENCODE data at the ENCODE portal. Nucleic Acids Res.

[50] Luo Y, Hitz BC, Gabdank I, Hilton JA, Kagda MS, Lam B (2020). New developments on the encyclopedia of DNA elements (ENCODE) data portal. Nucleic Acids Res.

[51] Dunham I, Kundaje A, Aldred SF, Collins PJ, Davis CA, Doyle F (2012). An integrated encyclopedia of DNA elements in the human genome. Nature.

[52] Bottomly D, Long N, Schultz AR, Kurtz SE, Tognon CE, Johnson K (2022). Integrative analysis of drug response and clinical outcome in acute myeloid leukemia. Cancer Cell.

[53] Huang R, Zhao L, Chen H, Yin RH, Li CY, Zhan YQ (2014). Megakaryocytic differentiation of K562 cells induced by PMA reduced the activity of respiratory chain complex IV. PLoS ONE.

[54] Ren JG, Seth P, Everett P, Clish CB, Sukhatme VP (2010). Induction of erythroid differentiation in human erythroleukemia cells by depletion of malic enzyme 2. PLoS ONE.

[55] Fanucchi S, Mhlanga MM (2017). Enhancer-derived lncRNAs regulate genome architecture: fact or fiction?. Trends Genet.

[56] Libbrecht MW, Noble WS (2015). Machine learning applications in genetics and genomics. Nat Rev Genet.

[57] Pang LR, Huang MX, Li H, Chen G, Zhong GP, Yao B (2020). LINC00707 accelerates the proliferation, migration and invasion of clear cell renal cell carcinoma. Eur Rev Med Pharmacol Sci.

[58] Constanty F, Shkumatava A (2021). lncRNAs in development and differentiation: from sequence motifs to functional characterization. Development.

[59] Dey BK, Mueller AC, Dutta A (2014). Long non-coding RNAs as emerging regulators of differentiation, development, and disease. Transcription.

[60] Liu Z, Zhang Y, Han X, Li C, Yang X, Gao J (2020). Identifying cancer-related lncRNAs based on a convolutional neural network. Front Cell Dev Biol.

[61] Shalem O, Sanjana NE, Zhang F (2015). High-throughput functional genomics using CRISPR-Cas9. Nat Rev Genet.

[62] Evers B, Jastrzebski K, Heijmans JPM, Grernrum W, Beijersbergen RL, Bernards R (2016). CRISPR knockout screening outperforms shRNA and CRISPRi in identifying essential genes. Nat Biotechnol.

[63] Lundberg SM, Allen PG, Lee S-I. A unified approach to interpreting model predictions. https://github.com/slundberg/shap. Accessed 4 Jan 2024.

[64] Franklin J (2005). The elements of statistical learning: data mining, inference and prediction. Math Intell.

[65] Altman N, Krzywinski M (2018). The curse(s) of dimensionality. Nat Methods.

[66] Nuñez JK, Chen J, Pommier GC, Cogan JZ, Replogle JM, Adriaens C (2021). Genome-wide programmable transcriptional memory by CRISPR-based epigenome editing. Cell.

[67] Dempster JM, Boyle I, Vazquez F, Root DE, Boehm JS, Hahn WC (2021). Chronos: a cell population dynamics model of CRISPR experiments that improves inference of gene fitness effects. Genome Biol.

[68] Cao C, Zhang T, Zhang D, Xie L, Zou X, Lei L (2017). The long non-coding RNA, SNHG6-003, functions as a competing endogenous RNA to promote the progression of hepatocellular carcinoma. Oncogene.

[69] Chen K, Wang X, Wei B, Sun R, Wu C, Yang H-J (2022). LncRNA SNHG6 promotes glycolysis reprogramming in hepatocellular carcinoma by stabilizing the BOP1 protein. Anim Cells Syst.

[70] Lu W, Cao F, Feng L, Song G, Chang Y, Chu Y (2021). LncRNA Snhg6 regulates the differentiation of MDSCs by regulating the ubiquitination of EZH2. J Hematol Oncol.

[71] Wang HS, Zhang W, Zhu HL, Li QP, Miao L, Miao L (2020). Long noncoding RNA SNHG6 mainly functions as a competing endogenous RNA in human tumors. Cancer Cell Int.

[72] Liu F, Tian T, Zhang Z, Xie S, Yang J, Zhu L (2022). Long non-coding RNA SNHG6 couples cholesterol sensing with mTORC1 activation in hepatocellular carcinoma. Nat Metab.

[73] Xu M, Chen X, Lin K, Zeng K, Liu X, Xu X (2019). LncRNA SNHG6 regulates EZH2 expression by sponging miR-26a/b and miR-214 in colorectal cancer. J Hematol Oncol.

[74] Lan Z, Yao X, Sun K, Li A, Liu S, Wang X (2020). The interaction between lncRNA SNHG6 and hnRNPA1 contributes to the growth of colorectal cancer by enhancing aerobic glycolysis through the regulation of alternative splicing of PKM. Front Oncol.

[75] Weng H, Huang H, Chen J (2019). RNA N 6-methyladenosine modification in normal and malignant hematopoiesis. Adv Exp Med Biol.

[76] Ferreira R, Ohneda K, Yamamoto M, Philipsen S (2005). GATA1 function, a paradigm for transcription factors in hematopoiesis. Mol Cell Biol.

[77] Zimta AA, Tomuleasa C, Sahnoune I, Calin GA, Berindan-Neagoe I (2019). Long non-coding RNAs in myeloid malignancies. Front Oncol.

[78] Gil N, Ulitsky I (2020). Regulation of gene expression by cis-acting long non-coding RNAs. Nat Rev Genet.

[79] Zou Q, Du X, Zhou L, Yao D, Dong Y, Jin J (2023). A short peptide encoded by long non-coding RNA small nucleolar RNA host gene 6 promotes cell migration and epithelial–mesenchymal transition by activating transforming growth factor-beta/SMAD signaling pathway in human endometrial cells. J Obstet Gynaecol Res.

