# Supplementary material for: Integration of transcription regulation and functional genomic data reveals lncRNA SNHG6’s role in hematopoietic differentiation and leukemia
Source: J Biomed Sci. 2024 Feb 28;31:27. doi: 10.1186/s12929-024-01015-8 (PMC10900714; doi:10.1186/s12929-024-01015-8)
Supplement: Supplementary file 1 — Additional file 1: Fig. S1. Building the machine learning algorithm. a Process followed for model training. The functional screening based on CRISPRi and the ENCODE Transcription Factor datasets were split into 90% for the training set (adopting a stratified cross-validation) and 10% for the testing set; along with binary labels indicating whether the lncRNA locus is either a hit or not hit. b XGBoost first residual-tree. Tree nodes are represented as rounded grey boxes, and squared white boxes are the tree leafs. c ROC curves comparing XGBoost, balanced random forest, and logistic regression models. d XGBoost (upper) and balanced random forest (lower) confusion matrices. Models were trained on 90% of the data, and ROC curves and confusion matrices show predictive value on the remaining 10%. Percentages from confusion matrices are row-normalized. e, f Under-sampling PCA. PCA of random under-sampling of the majority class (i.e. not hit) without (e) and with (f) replacement, plotting the complete dataset (upper-left plot) plus 8 sampling strategies. PCA values based on 130 numeric features showing the removed not hit transcripts. Red dots: hit; grey dots: not hit. g Recursive feature elimination. Iteratively, one feature was removed to train a new model, removing the least important. Red and black lines denote the optimal number of features (n = 71) and sensitivity value using 143 features, respectively. h SHAP dependence plot for the TSS PC distance feature. Each blue dot denotes a lncRNA. Positive odd values (above dashed line) contribute towards prediction of hits. i Detailed explanation for the INFLAMeR score of SNHG6. The final INFLAMeR score of SNHG6 was 0.504. Fig. S2. sgRNA control samples show distinct clustering after batch effect correction. After batch effect correction using the NOISeq package, the samples were clustered according to the target KD; however, the sgRNA control samples were distinctly clustered from all lncRNA KD samples. Fig. S3. Confirming [file 12929_2024_1015_MOESM1_ESM.pdf]

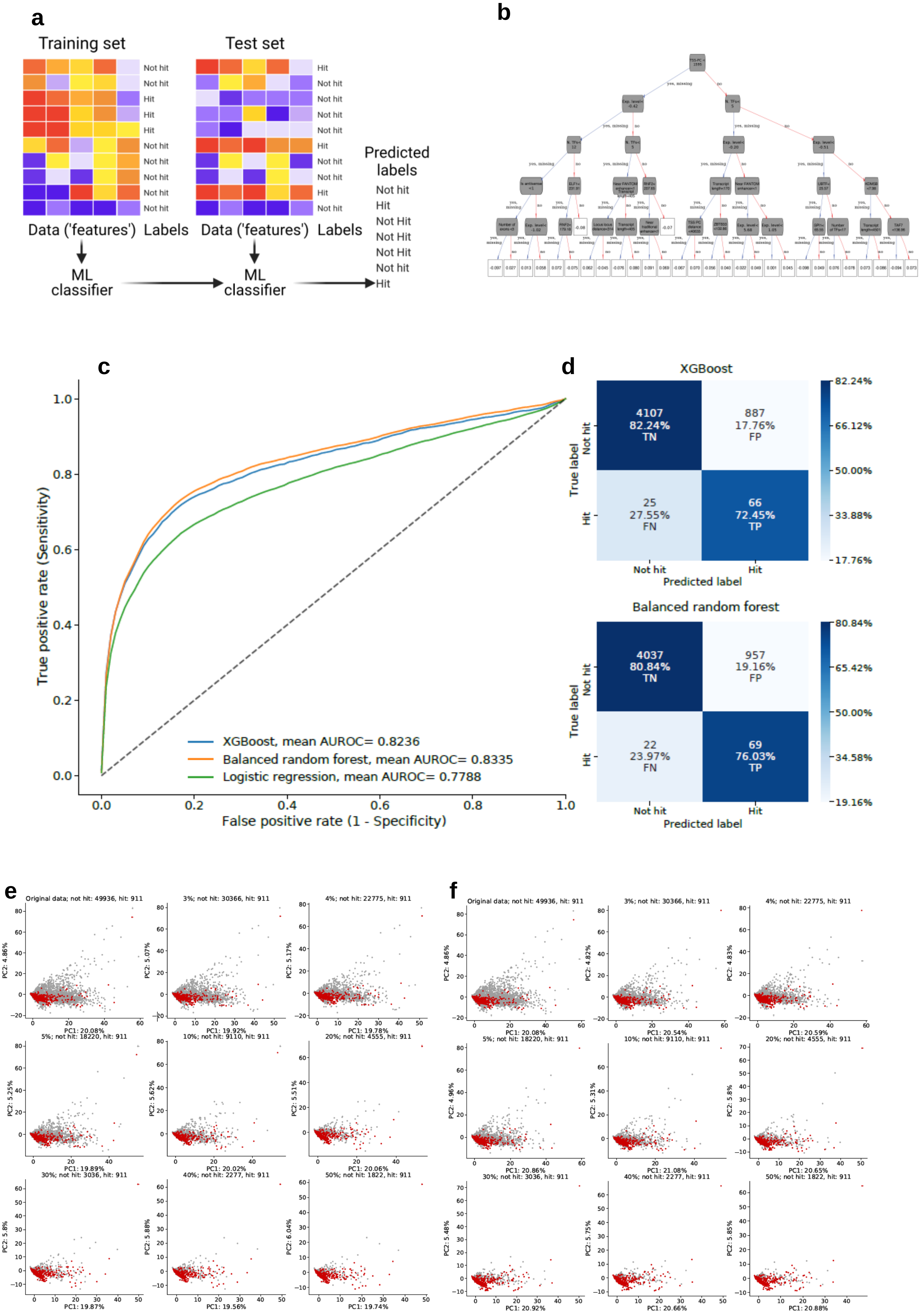
a
Training set
Test set
Not hit
Hit
Predicted labels
Data ('features')
Labels
ML classifier
b
c
True positive rate (Sensitivity)
False positive rate (1 - Specificity)
XGBoost, mean AUROC= 0.8236
Balanced random forest, mean AUROC= 0.8335
Logistic regression, mean AUROC= 0.7788
d
XGBoost
4107
82.24%
TN
887
17.76%
FP
25
27.55%
FN
66
72.45%
TP
True label
Predicted label
Balanced random forest
4037
80.84%
TN
957
19.16%
FP
22
23.97%
FN
69
76.03%
TP
e
Original data; not hit: 49936, hit: 911
3%; not hit: 30366, hit: 911
4%; not hit: 22775, hit: 911
5%; not hit: 18220, hit: 911
10%; not hit: 9110, hit: 911
20%; not hit: 4555, hit: 911
30%; not hit: 3036, hit: 911
40%; not hit: 2277, hit: 911
50%; not hit: 1822, hit: 911
f

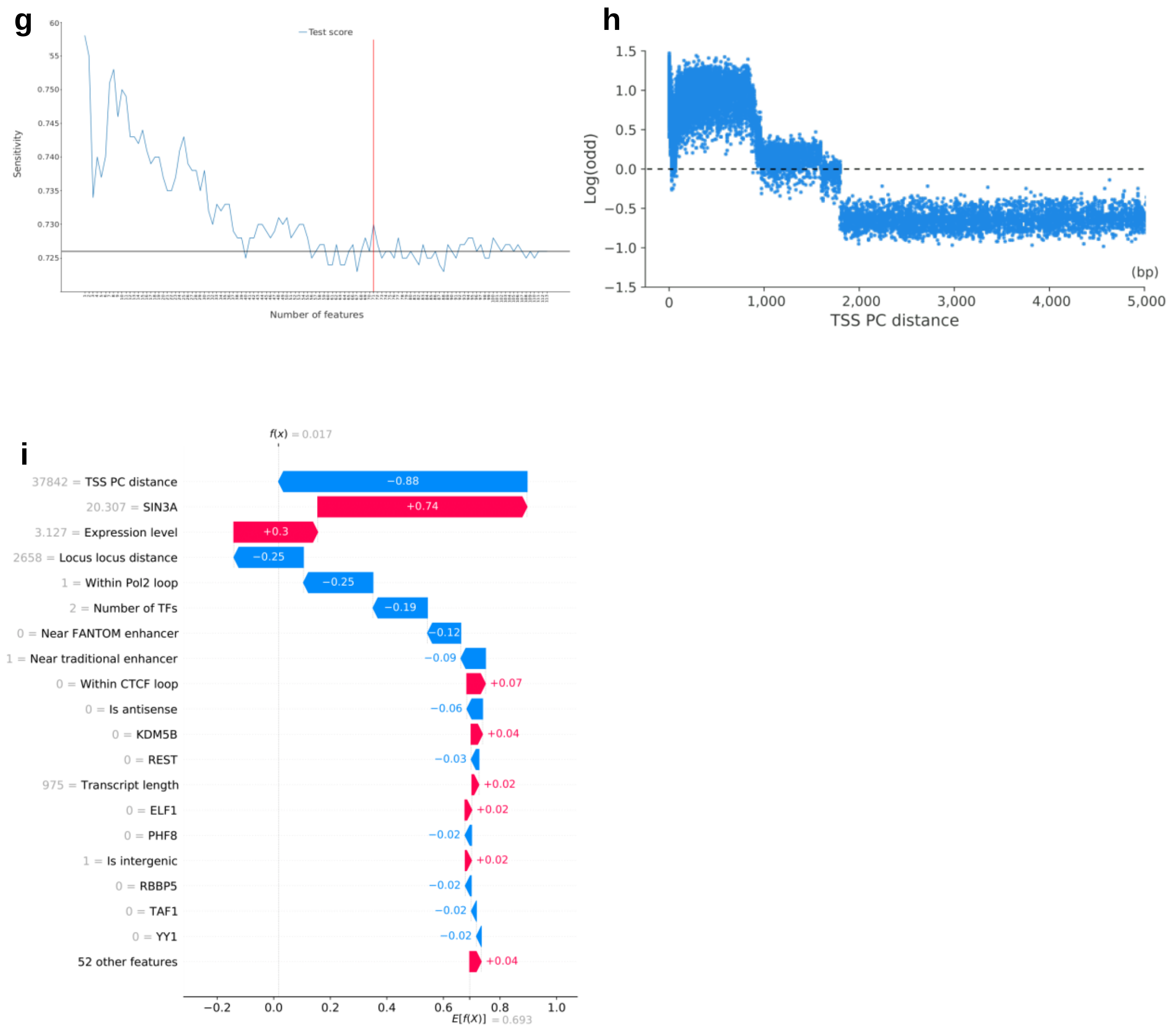

Supplemental Fig. S1. Building the machine learning algorithm. (a) Process followed for model training. The functional screening based on CRISPRi and the ENCODE Transcription Factor datasets were split into 90% for the training set (adopting a stratified cross-validation) and 10% for the testing set; along with binary labels indicating whether the lncRNA locus is either a hit or not hit. (b) XGBoost first residual-tree. Tree nodes are represented as rounded grey boxes, and squared white boxes are the tree leafs. (c) ROC curves comparing XGBoost, balanced random forest, and logistic regression models. (d) XGBoost (upper) and balanced random forest (lower) confusion matrices. Models were trained on 90% of the data, and ROC curves and confusion matrices show predictive value on the remaining 10%. Percentages from confusion matrices are row-normalized. (e, f) Under-sampling PCA. PCA of random under-sampling of the majority class (i.e. not hit) without (e) and with (f) replacement, plotting the complete dataset (upper-left plot) plus 8 sampling strategies. PCA values based on 130 numeric features showing the removed not hit transcripts. Red dots: hit; grey dots: not hit. (g) Recursive feature elimination. Iteratively, one feature was removed to train a new model, removing the least important. Red and black lines denote the optimal number of features (n = 71) and sensitivity value using 143 features, respectively. (h) SHAP dependence plot for the TSS PC distance feature. Each blue dot denotes a lncRNA. Positive odd values (above dashed line) contribute towards prediction of hits. (i) Detailed explanation for the INFLAMeR score of SNHG6. The final INFLAMeR score of SNHG6 was 0.504.

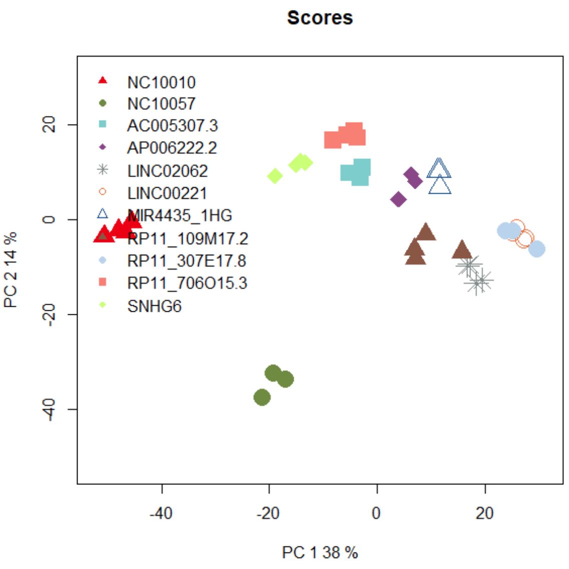

Supplemental Fig. S2. sgRNA control samples show distinct clustering after batch effect correction. After batch effect correction using the NOISeq package, the samples were clustered according to the target KD; however, the sgRNA control samples were distinctly clustered from all lncRNA KD samples.

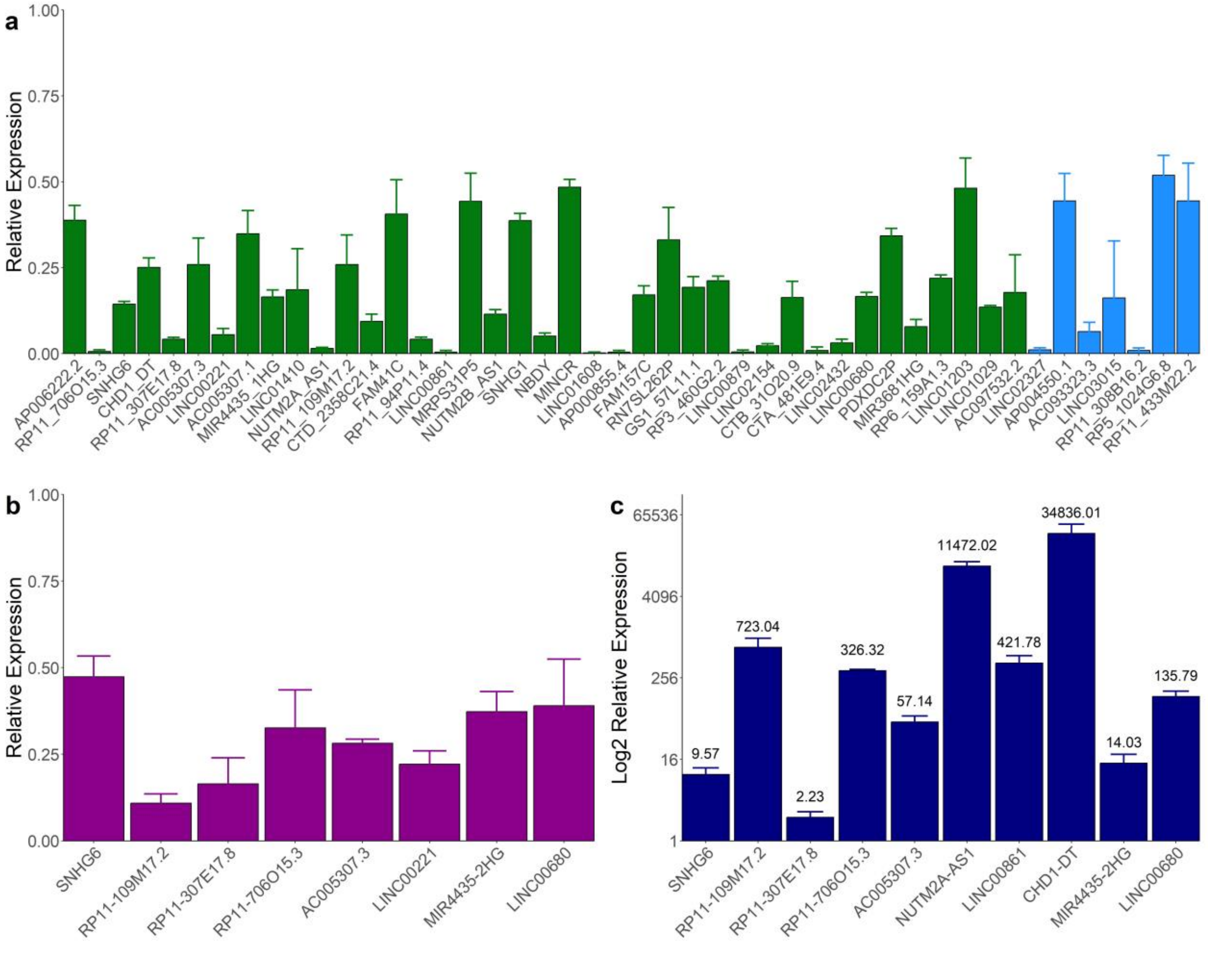

Supplemental Fig. S3. Confirming the change in lncRNA expression after knockdown (KD), knockout (KO), and overexpression by qPCR. (a) KD of thirty-nine lncRNAs predicted to be functional (red) and seven lncRNAs predicted to be non-functional (blue). (b) KO of eight top performing lncRNAs. (c) Confirming overexpression of ten top performing lncRNAs following stable transduction of a lentiviral plasmid containing the lncRNA sequence. Expression is given relative to that in samples transduced with a non-targeting control sgRNA.

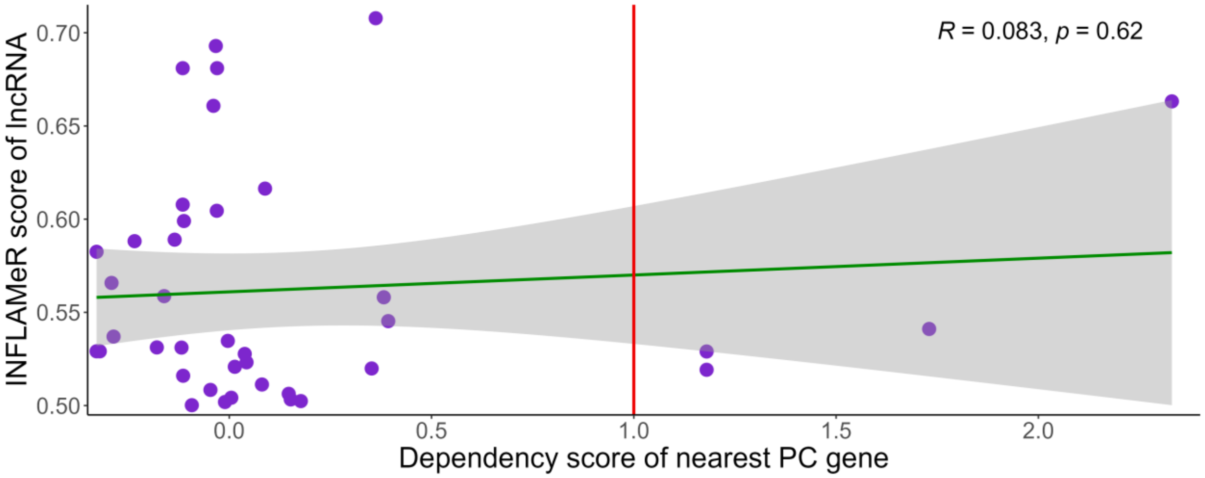

Supplemental Fig. S4. There was no correlation between INFLAMeR score and the essentiality of neighboring protein-coding (PC) genes. Genes with a dependency score > 1 (red line) are considered essential. Green line represents the linear regression with 95% confidence interval (grey).

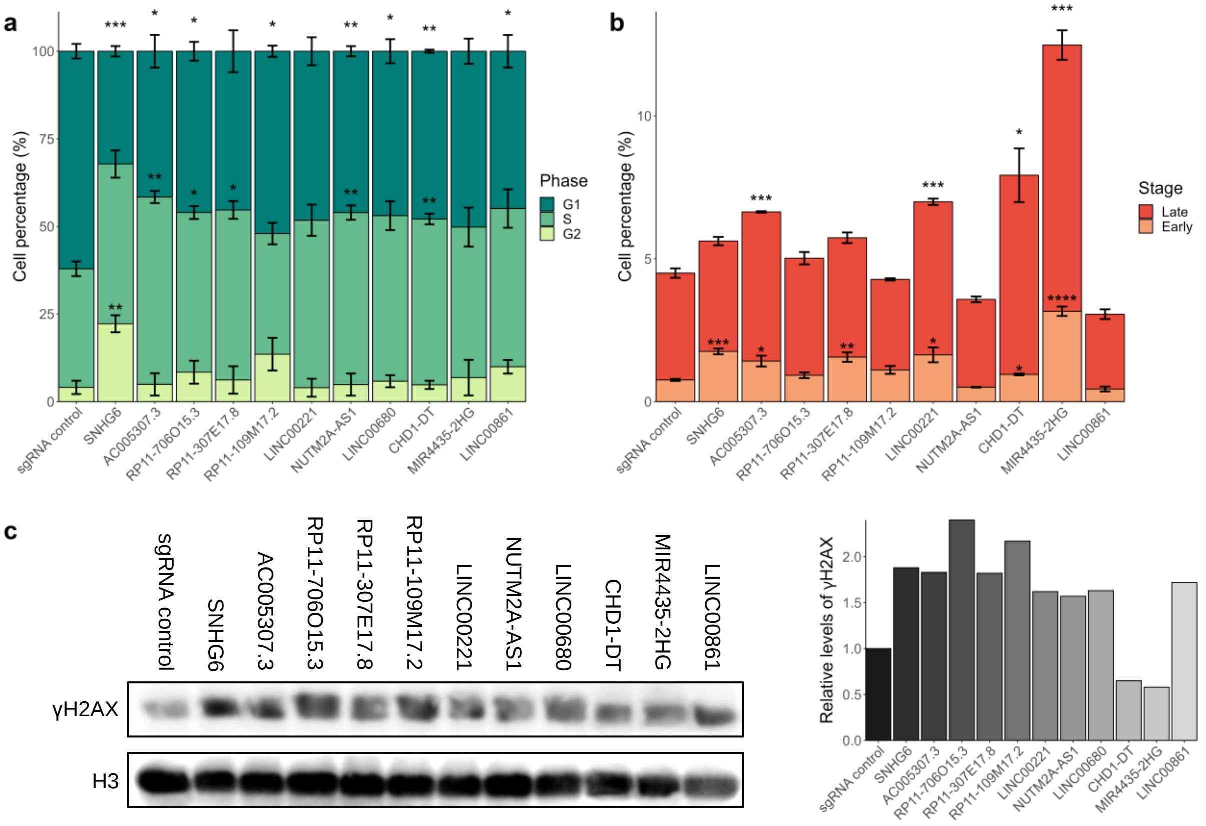

Supplemental Fig. S5. Knockdown of top performing lncRNAs affects cell cycle, apoptosis, and DNA damage. Knockdown of the indicated lncRNAs caused dysregulation of the cell cycle (a), increased apoptosis rates (b), and increased rates of DNA damage (c), as indicated by increased levels of γH2AX. * $p < 0.05$, ** $p < 0.01$, *** $p < 0.001$, **** $p < 0.0001$ vs. sgRNA control (n = 3).

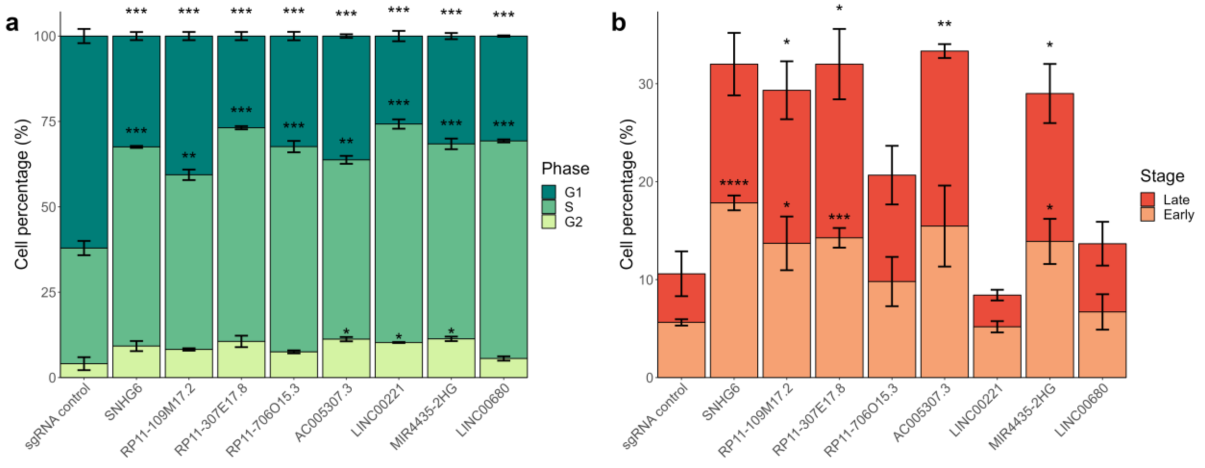

Supplemental Fig. S6. Knockout of top performing lncRNAs affects cell cycle and apoptosis. Functional knockout of the indicated lncRNAs by TSS deletion caused dysregulation of the cell cycle (a) and increased apoptosis rates (b), replicating the results seen after knockdown (See Supp. Fig. S4). * $p < 0.05$, ** $p < 0.01$, *** $p < 0.001$, **** $p < 0.0001$ vs. sgRNA control (n = 3).

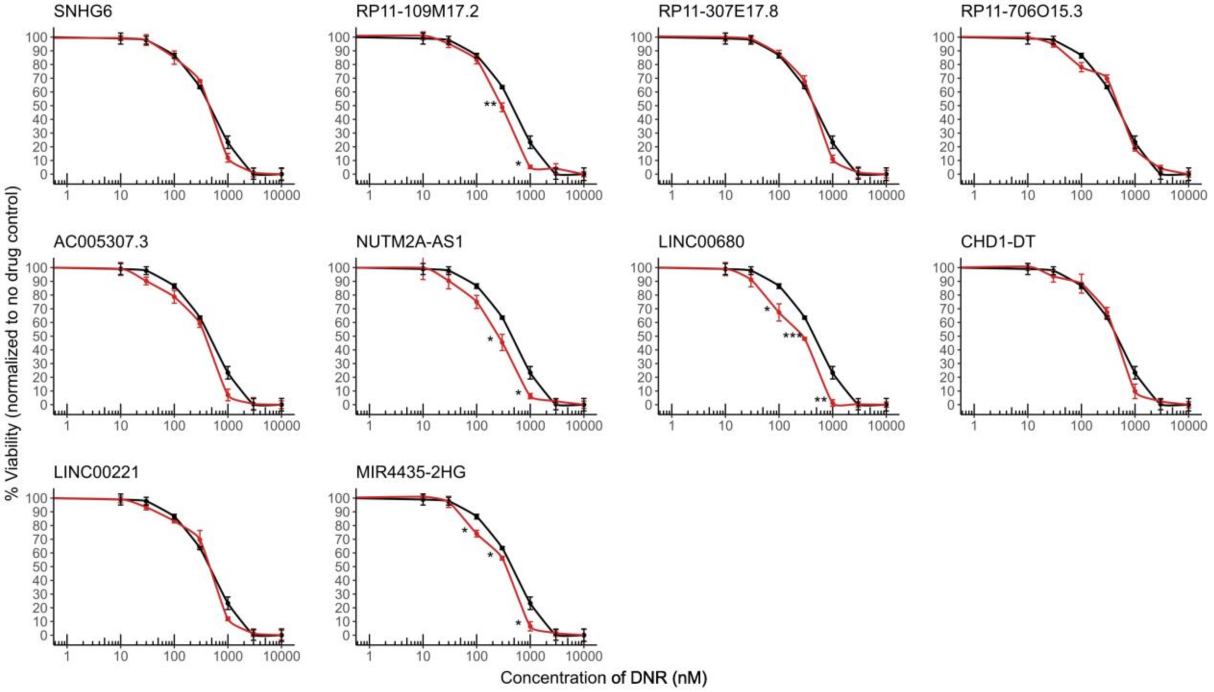

Supplemental Fig. S7. Determining the IC50 for daunorubicin (DNR) of selected samples. K562 cells from the indicated samples were incubated with the indicated concentrations of DNR for 72 h and their viability was determined using the XTT assay relative to that in untreated cells. Values are given as the mean ± SD for n = 3 biological replicates. The black line in each curve represents the non-targeting sgRNA control sample. * $p < 0.05$, ** $p < 0.01$, *** $p < 0.001$ vs. sgRNA control.

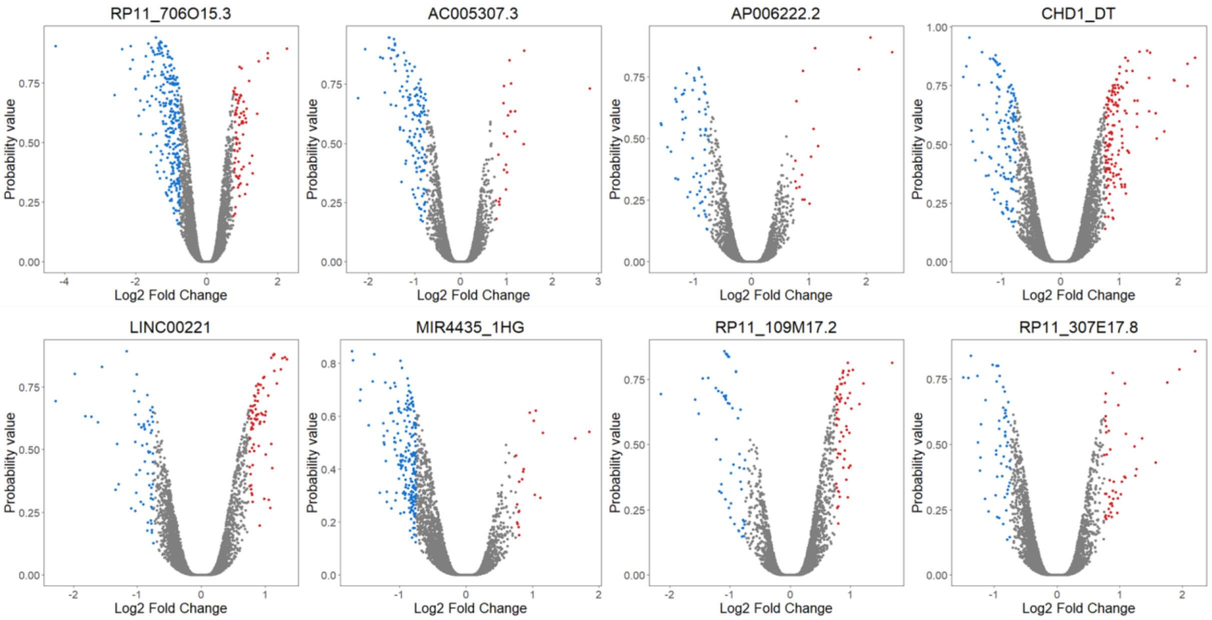

Supplemental Fig. S8. Differentially expressed genes from each sample. KD of the indicated lncRNAs generally led to a higher proportion of downregulated genes (blue) compared to upregulated genes (red).

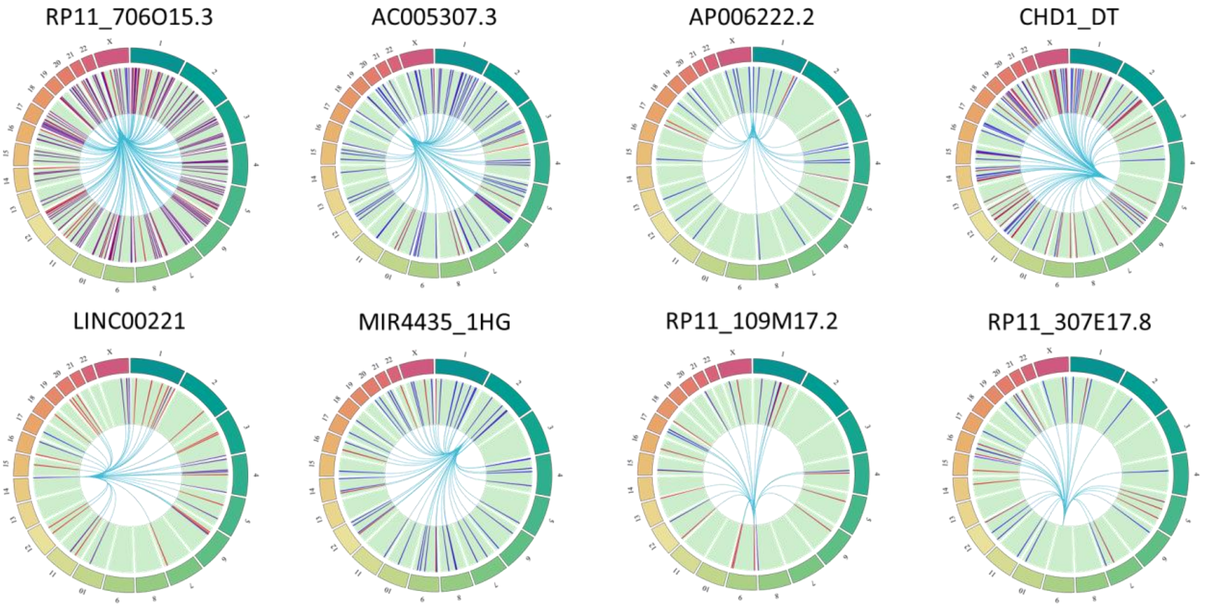

Supplemental Fig. S9. KD of the indicated lncRNAs affected the expression of genes across the genome. Red represents upregulation, blue represents downregulation.

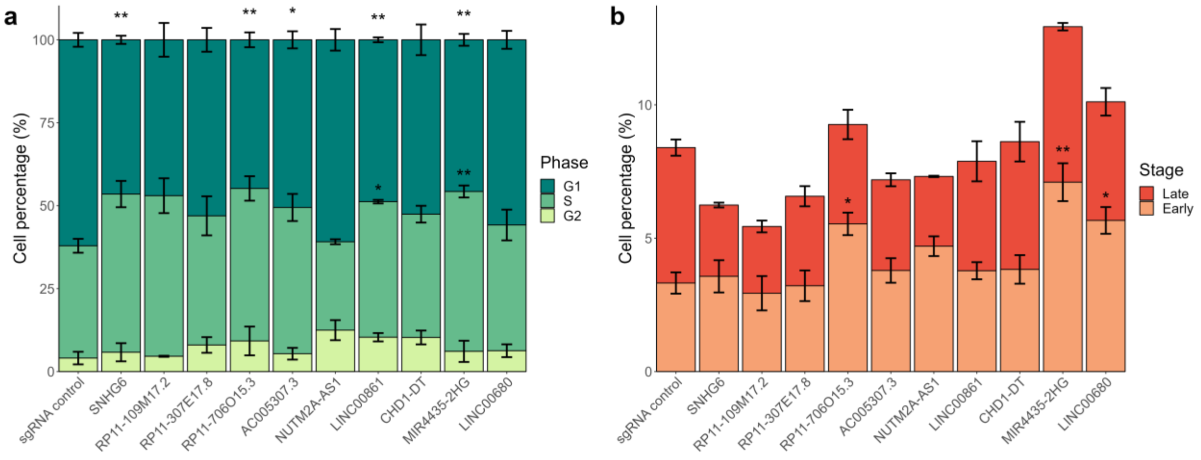

Supplemental Fig. S10. Overexpression of top performing lncRNAs rescued cell cycle regulation and apoptosis rates. Rescuing the expression of the indicated lncRNAs after knockdown partially or fully restored cell cycle regulation (a) and reduced apoptosis rates (b) for most of the samples (See Supp. Fig. S4). * *p* < 0.05, ** *p* < 0.01 vs. sgRNA control (n = 3).

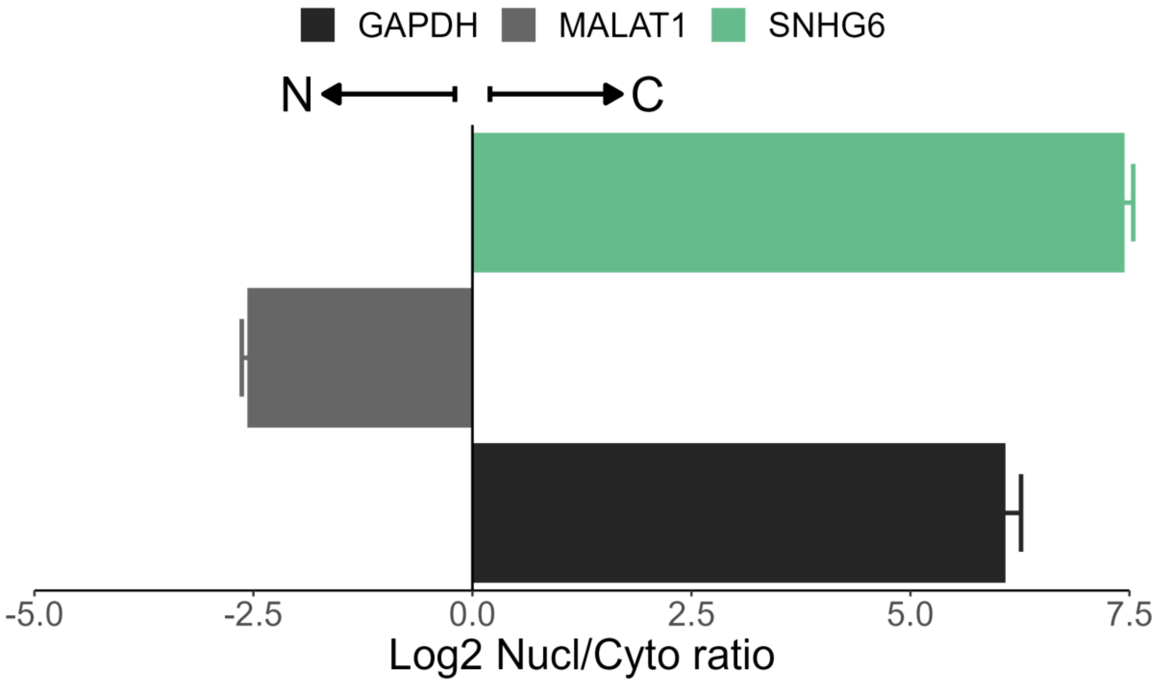

Supplemental Fig. S11. SNHG6 subcellular localization. The enrichment of SNHG6 was measured by qPCR in the nuclear/cytoplasmic fractions of K562 cells. MALAT1 and GAPDH were used as nuclear and cytoplasmic controls, respectively.

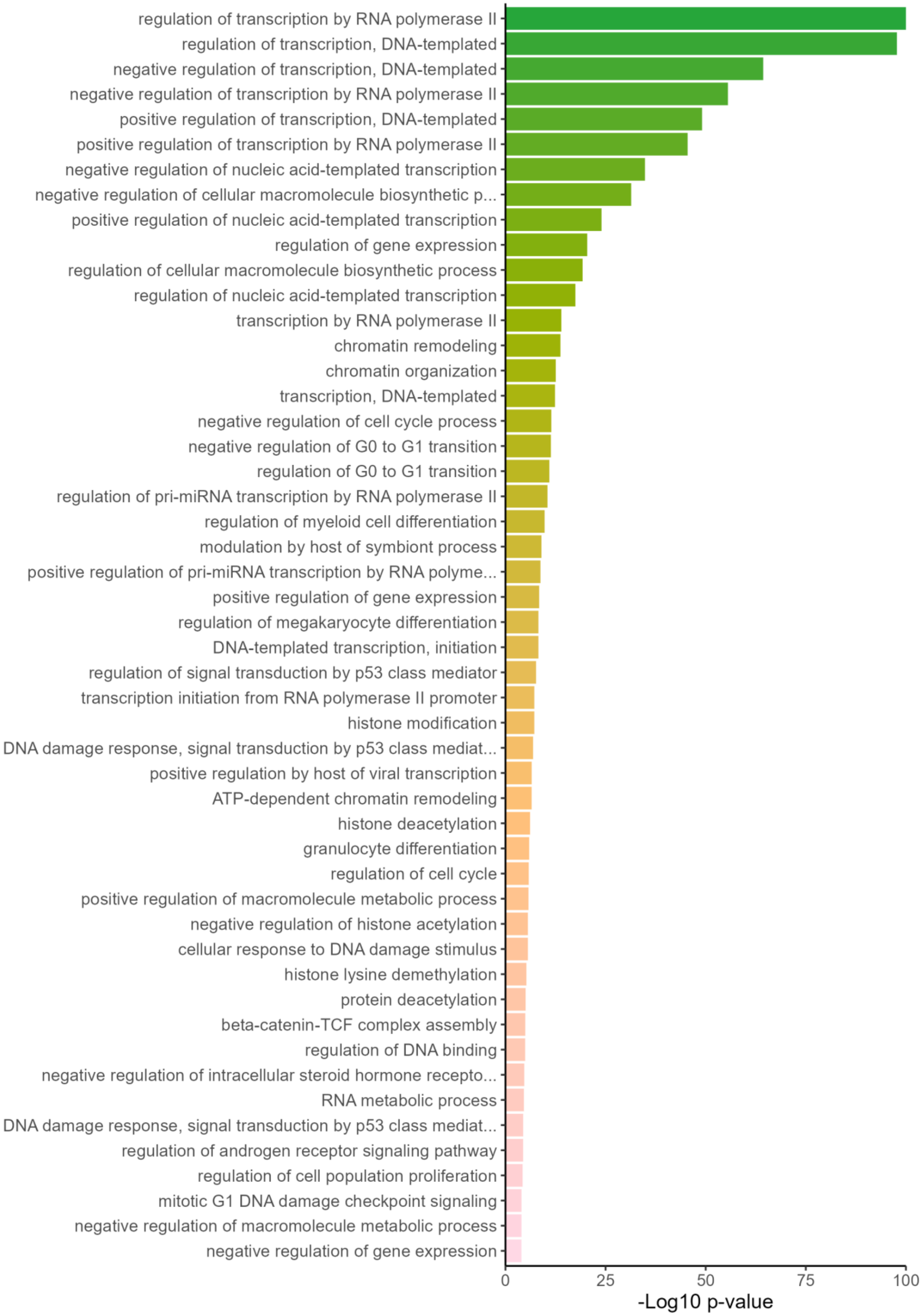

Supplemental Fig. S12. Gene ontology analysis for the transcription factors that bind the promoter of SNHG6 in K562. The top 50 pathways are shown.

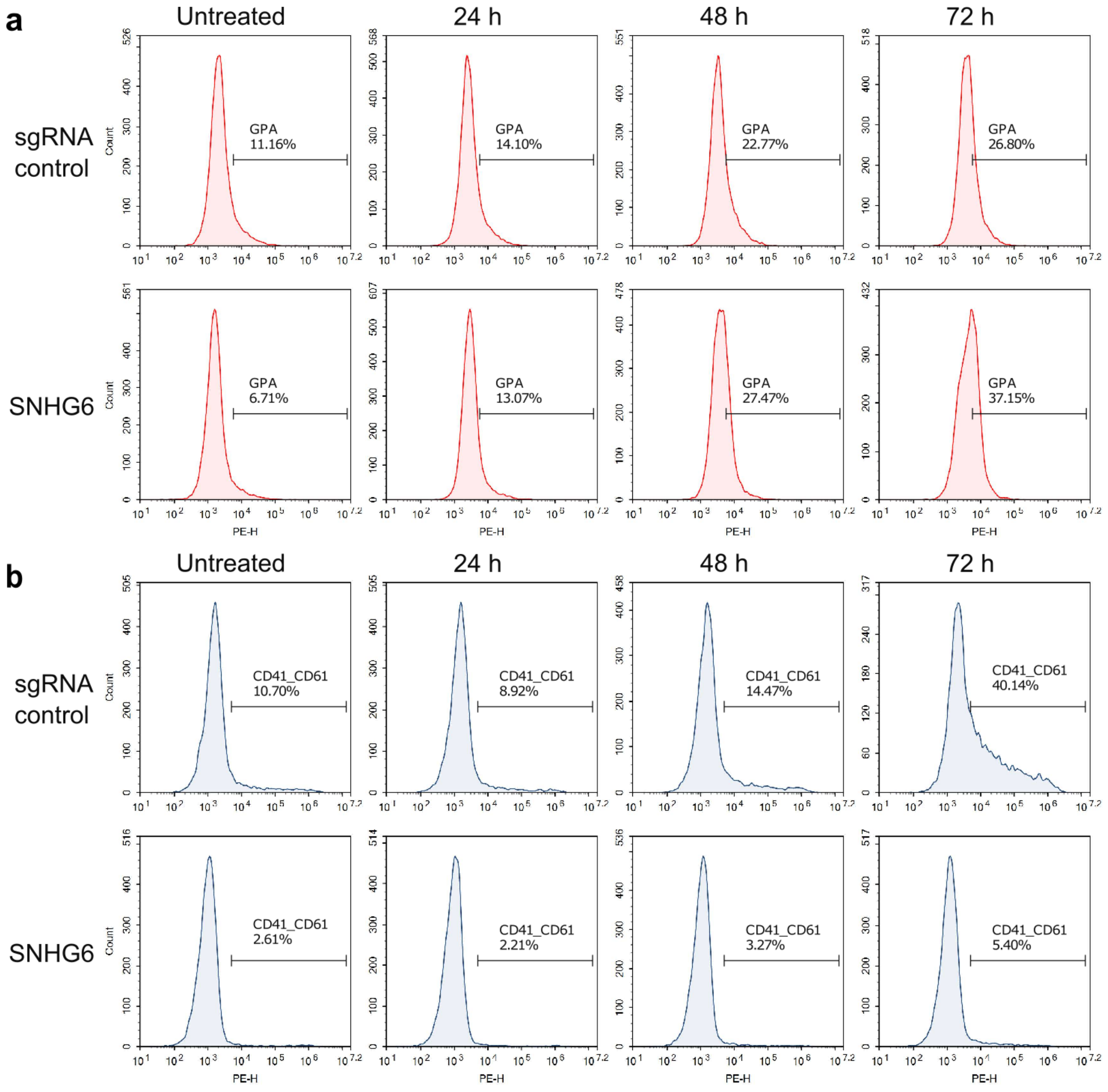

Supplemental Fig. S13. Measuring the levels of myeloid differentiation markers by flow cytometry. (a) Erythrocyte differentiation was assessed based on glycophorin A (GPA) levels using immunostaining with flow cytometry in cells incubated with 30 µM hemin for 72 h. (b) Megakaryocyte differentiation was measured based on CD41/CD61 levels using immunostaining with flow cytometry in cells incubated with 0.2 nM PMA for 72 h.

Supplemental Table S5. The 143 features included in the initial algorithm.

Log2 FPKM	Is intergenic	Is antisense	TSS PC distance	Locus-locus distance	Locus is heterozygous deleted	Locus is amplified	Near cancer associated SNP	Near fantom enhancer
Near hnisz super enhancer	Near hnisz enhancer	Within CTCF loop	Within Pol2 loop	Transcript length	Number of exons	Has mouse ortholog	Number of TFs	ATF1
ATF2	CBX3	CEBPB	CHD2	CREB1	CTCF	E2F1	E2F6	EGR1
ELF1	EP300	ESRRA	FOS	FOSL1	FOXM1	GABPA	GTF2F1	HCFC1
HDAC1	IKZF1	IRF1	JUND	KDM5B	MAFF	MAFK	MAZ	MTA3
MYC	NCOR1	NFIC	NR2C2	PHF8	POLR2A	RAD21	RBBP5	REST
RNF2	SAP30	SIN3A	SMARCA4	SP1	SPI1	SREBF2	TAF1	TAF7
TBP	TEAD4	THAP1	UBTF	YY1	ZBTB33	ZBTB7A	ZNF384	
Locus is homozygous deleted	*Near vista enhancer*	*ARID3A*	*ATF3*	*BACH1*	*BCLAF1*	*BHLHE40*	*BRCA1*	*CBX8*
CEBPZ	*CHD1*	*CHD7*	*CTBP2*	*CTCFL*	*CUX1*	*E2F4*	*ELK1*	*ETS1*
EZH2	*FOSL2*	*FOXA1*	*GATA1*	*GATA2*	*GATA3*	*HDAC2*	*HDAC6*	*HSF1*
JUN	*KDM1A*	*KDM5A*	*MAX*	*MEF2A*	*MXI1*	*MYBL2*	*NANOG*	*NFE2*
NFYA	*NFYB*	*NR2F2*	*NR3C1*	*NRF1*	*PML*	*POU5F1*	*RCOR1*	*RELA*
RFX5	*RXRA*	*SETDB1*	*SIX5*	*SMARCB1*	*SMARCC2*	*SMC3*	*SREBF1*	*SRF*
STAT5A	*SUPT20H*	*SUZ12*	*TAL1*	*TBL1XR1*	*TCF12*	*TCF7L2*	*TRIM28*	*USF1*
USF2	*ZC3H11A*	*ZKSCAN1*	*ZMIZ1*	*ZNF143*	*ZNF217*	*ZNF263*	*ZNF274*	*ZZZ3*

Italics: not included in the final algorithm.

Supplemental Table S6. Cost-sensitive model metrics.

Model	Sensitivity	Specificity	AUROC	F1	Precision	Brier score	Training time (s)
XGBoost	0.7245	0.8224	0.8236	0.1264	0.0693	0.1638	54.7
Balanced random forest	0.7603	0.8084	0.8335	0.1240	0.0675	0.1460	73.0
Logistic regression	0.6165	0.8569	0.7788	0.1304	0.0629	0.1442	58.1

Values based on the mean of three randomization seeds of the test set.

Supplemental Table S7. Under-sampling strategies without replacement.

Sampling strategy	Sensitivity	Specificity	AUROC	F1	Precision
3%	0.1627	0.9961	0.8250	0.2360	0.4363
4%	0.2001	0.9942	0.8270	0.2621	0.3868
5%	0.2341	0.9924	0.8281	0.2818	0.3583
10%	0.3556	0.9826	0.8270	0.3073	0.2716
20%	0.4946	0.9588	0.8292	0.2633	0.1797
30%	0.5638	0.9342	0.8302	0.2182	0.1354
40%	0.6114	0.9111	0.8289	0.1888	0.1117
50%	0.6458	0.8894	0.8269	0.1679	0.0966

Preprocessing sampling strategies applied before XGBoost training.

Supplemental Table S8. Under-sampling strategies with replacement.

Sampling strategy	Sensitivity	Specificity	AUROC	F1	Precision
3%	0.1895	0.9945	0.8238	0.2531	0.3858
4%	0.2301	0.9928	0.8257	0.2815	0.3665
5%	0.2594	0.9906	0.8258	0.2918	0.3356
10%	0.3604	0.9807	0.8307	0.2980	0.2546
20%	0.4873	0.9589	0.8316	0.2609	0.1784
30%	0.5675	0.9337	0.8301	0.2183	0.1353
40%	0.6172	0.918	0.8306	0.1915	0.1134
50%	0.6312	0.8897	0.8253	0.1646	0.0947

Preprocessing sampling strategies applied before XGBoost training.

Supplemental Table S9. Model performance comparison.

Model	Sensitivity	Specificity	AUROC	F1	Precision	Brier score
Cost-sensitive XGBoost 71 features	0.7292	0.8227	0.8250	0.1275	0.0698	0.1634
Cost-sensitive XGBoost 143 features	0.7245	0.8224	0.8236	0.1264	0.0693	0.1638
Balanced random forest 143 features	0.7603	0.8084	0.8335	0.1240	0.0675	0.1460
Cost-sensitive logistic regression 143 features	0.6165	0.8569	0.7788	0.1304	0.0729	0.1442
Under-sampling XGBoost without replacement 143 features	0.6484	0.8894	0.8267	0.1679	0.0966	0.0907
Under-sampling XGBoost with replacement 143 features	0.6312	0.8897	0.8249	0.1646	0.0947	0.0915

Metrics based on the mean of 3 randomization seeds of the test set.

Supplemental Table S10. Performance of 10-fold cross-validation (CV).

10-fold CV	AUROC-1	AUROC-2	AUROC-3
1	0.78	0.80	0.80
2	0.82	0.85	0.84
3	0.83	0.82	0.82
4	0.83	0.80	0.82
5	0.79	0.85	0.82
6	0.83	0.82	0.83
7	0.82	0.85	0.85
8	0.83	0.82	0.82
9	0.84	0.85	0.84
10	0.87	0.84	0.81

Each column represents one randomization seed.
